# Transcriptomes of parents identify parenting strategies and sexual conflict in a subsocial beetle

**DOI:** 10.1038/ncomms9449

**Published:** 2015-09-29

**Authors:** Darren J. Parker, Christopher B. Cunningham, Craig A. Walling, Clare E. Stamper, Megan L. Head, Eileen M. Roy-Zokan, Elizabeth C. McKinney, Michael G. Ritchie, Allen J. Moore

**Affiliations:** 1 Centre for Biological Diversity, School of Biology, University of St Andrews, Fife KY16 9TH, UK; 2 Department of Biological and Environmental Science, University of Jyväskylä, Jyväskylä 40014, Finland; 3 Department of Genetics, University of Georgia, Athens, Georgia 30602, USA; 4 Centre for Ecology and Conservation, College of Life and Environmental Sciences, University of Exeter, Penryn TR10 9EZ, UK; 5 Institute of Evolutionary Biology, School of Biological Sciences, University of Edinburgh, Edinburgh EH9 3JT, UK; 6 Division of Evolution, Ecology and Genetics, Research School of Biology, The Australian National University, Canberra, Australian Capital Territory 0200, Australia

## Abstract

Parenting in the burying beetle *Nicrophorus vespilloides* is complex and, unusually, the sex and number of parents that can be present is flexible. Such flexibility is expected to involve specialized behaviour by the two sexes under biparental conditions. Here, we show that offspring fare equally well regardless of the sex or number of parents present. Comparing transcriptomes, we find a largely overlapping set of differentially expressed genes in both uniparental and biparental females and in uniparental males including *vitellogenin*, associated with reproduction, and *takeout*, influencing sex-specific mating and feeding behaviour. Gene expression in biparental males is similar to that in non-caring states. Thus, being ‘biparental’ in *N. vespilloides* describes the family social organization rather than the number of directly parenting individuals. There was no specialization; instead, in biparental families, direct male parental care appears to be limited with female behaviour unchanged. This should lead to strong sexual conflict.

## Abstract (web summary)

The burying beetle shows flexible parenting behaviour. Here, the authors show that offspring fare equally well regardless of the sex or number of parents present and find similar gene expression profiles in uniparental and biparental females and in uniparental males, which suggests no specialization in parenting.

## Graphical abstract

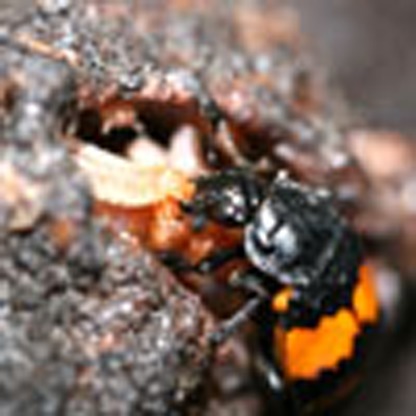

Parental care, although relatively unusual, is taxonomically widespread and surprisingly diverse^1^,^2^. Most species that care for their offspring exhibit uniparental care and, except in fish, it is typically the female that provides the care^3^,^4^. Yet biparental care can be the ancestral condition^4^,^5^. We still do not know why biparental care is relatively rare or why females are typically the caring parent given both parents benefit from the care provided to their offspring. It is also possible that the presence of both parents does not mean that both actively care for the offspring. Biparental social conditions therefore describe the presence of two (potential) parents, while biparental care describes the active participation of both in parenting the offspring. Theoretically, a biparental social condition with biparental care may be stable if one parent cannot successfully raise offspring alone^6^ or if biparental care can increase offspring fitness more than the fitness gained by the deserting parent via remating or increased survival^2^. It has been suggested that stable biparental care can also be maintained via specialization of parenting tasks by the sexes^7^. Potential for future reproduction^6^, certainty of paternity^8^, life-history differences^9^,^10^ and conflict between the sexes^11^ are all predicted to influence which sex cares. Despite this considerable theoretical work, experimental tests of the different models have provided ambiguous results. Biparental care does not always provide benefits over uniparental care even when it is the norm, and species exist that exhibit all forms of care within a population^12^,^13^.

Although most species are invariant in parenting strategies^6^, studying species where there is natural variation in both the social conditions (biparental or uniparental) and which parent(s) care provides an opportunity to examine potential fitness differences arising from different forms of care. Variable care also allows us to ask whether parenting strategies differ when an individual cares uniparentally or biparentally. Is care expressed in a biparental family the same as that expressed uniparentally? Are the sexes the same in how they parent alone and together? Behavioural studies by themselves can be inconclusive. Including studies of proximate genetic mechanisms involved in parenting with an examination of fitness effects is one way to overcome the limitation of behavioural studies^14^. Unfortunately, the genetic basis of parenting behaviour is poorly understood, except in model systems^15^,^16^. Examination of gene expression changes under different parental care strategies will allow us to assess similarities or differences in the genetic influences on parenting strategies^17^, and may also permit the detection of otherwise cryptic phenotypic differences^18^,^19^.

Here we have two objectives. First, we wished to determine the extent to which flexible parenting strategies differ in success in the burying beetle, *Nicrophorus vespilloides*, where both uniparental and biparental families occur. Second, we sought to determine the extent to which the sexes specialized in parenting strategies. We examined the outcomes of flexibility by measuring the fitness consequences of different forms of care when individuals were free to adopt the form of care provided. We tested for specialization by examining transcriptomic differences underlying the different care strategies when these were imposed on individuals. *N. vespilloides* breed on the carcasses of small vertebrates and directly provision begging young by regurgitating pre-digested carrion (Fig. 1). In addition, parents prevent a build-up of fungus and bacteria, and defend the carcass from predators and congeneric competitors^12^,^20^,^21^. Previous research has suggested that there may be specialization of the form of care provided, particularly by males, which increase interactions with the offspring when females are removed^22^,^23^. Females appear to be unaffected by the presence or absence of males. Experiments have also found no obvious fitness advantage to biparental care over uniparental care^22^,^23^,^24^,^25^. However, the extent of specialization observed in previous studies was relatively small and restricted to males, making it difficult to assess its importance in *N. vespilliodes*. A particular complication is that parental care is a complex phenotype and even detailed observational studies may fail to accurately measure the full repertoire of behaviours involved. We adopted an RNA-seq approach to examine the changes in gene expression in brains and associated tissues associated with parenting to capture signatures of specialization. Previous studies investigating a range of complex behaviours have demonstrated that gene expression assays are an effective way of identifying potentially coordinated changes in suites of genes associated with discrete behavioural states and identifying mechanistic pathways involved^18^,^19^. By using transcriptomics, we can identify which genes change expression when parenting, sex differences in such genes and whether sex-specific changes occur when parents are in isolation or biparental. For example, if sexes have specialized parental roles when together, we might expect male and female gene expression to differ more when providing care biparentally than when alone.

We report two experiments that identify differences in the family organization and functional parenting roles of males and females. We first allowed pairs of beetles to freely adopt a family social condition either as uniparental female, uniparental male or biparental pair. Previous studies have experimentally allocated individuals (by preventing or enforcing male or female dispersing under) but it is possible that inadvertent incompatible pairings or sexual conflict^11^,^26^ arising from enforced parings reduce benefits of biparental care for offspring. We show that biparental care is common but there is no detectable difference in fitness associated with number or sex of parent. Next we measured gene expression of both males and females under biparental and uniparental family social conditions: (1) before parenting, (2) actively parenting and (3) post parenting. By examining the changes in gene expression, we identify an overlapping set of genes associated with parenting behaviour in males and in females. We find that gene expression patterns are the same in uniparental males and females, and include genes such as *vitellogenin*, more commonly associated with reproduction in females, and *takeout*, associated with courtship in males and circadian-influenced feeding. Biparental males do not differentially express genes associated with parenting to the same extent as females, suggesting there should be a strong conflict between the sexes over parenting. We also find that gene expression patterns of post-caring individuals return to a pre-caring state in all social contexts, confirming the flexibility of parenting behaviour.

## Results

### Parental care and offspring fitness

**Figure 2:**
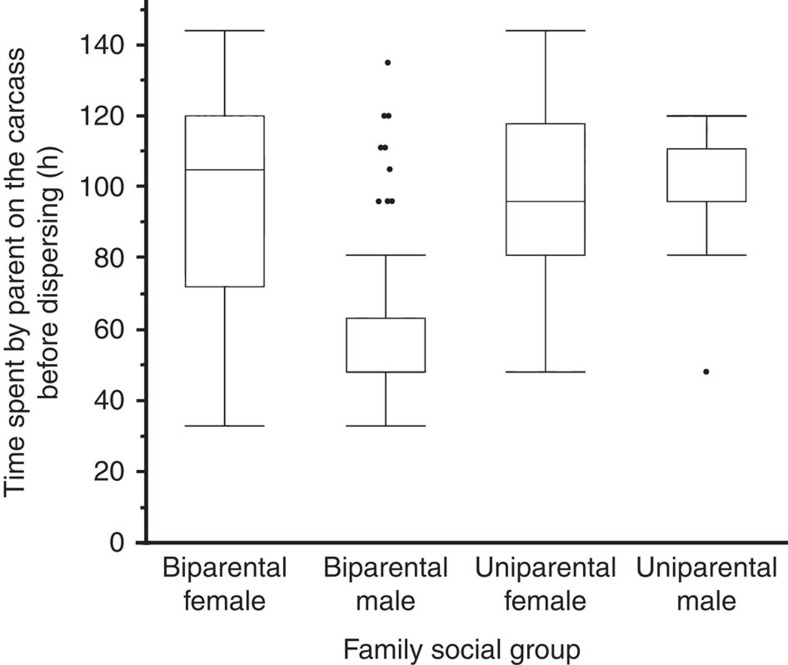
Variation in duration of parents remaining with the young under different forms of parental care. Duration of time spent on a carcass with offspring when individuals are free to adopt either uniparental or biparental family social condition. Data are presented using box plots, with medians (inner line; where there is no inner line, the median and 25% quartile overlap), 25 and 75% quartiles (boxes) and whiskers (end of box to 1.5 × interquartile range). This illustrates the extent of variation in different social groupings. The dots are values that fall outside the interquartile range, and show that males that adopt a biparental condition have more extreme values in the duration they remain on the carcass than are uniparental males or females in either uniparental or biparental conditions. Analyses are based on 138 biparental females and biparental males, 119 uniparental females and 13 uniparental males.

**Figure 3:**
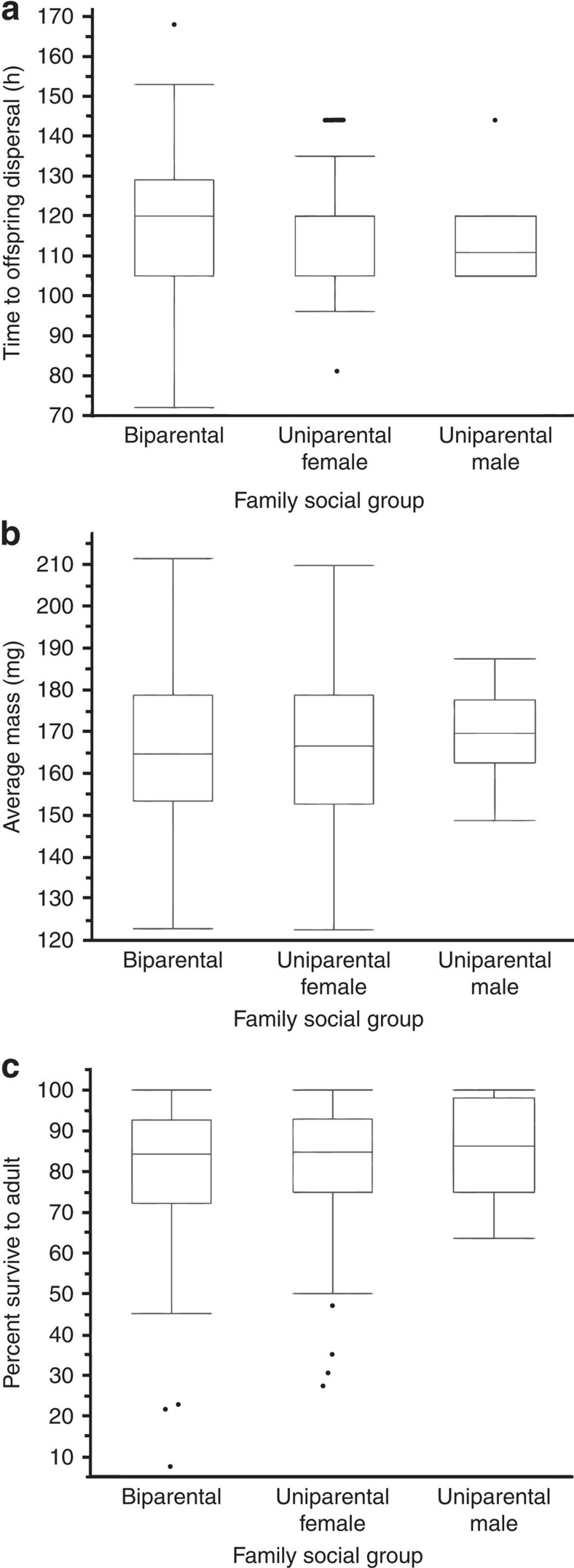
Performance and fitness measures of offspring under different family social conditions. (**a**) The time to dispersal of offspring from the carcass, measured in hours (h) from egg hatching. All offspring disperse together so there is a single dispersal time per brood. Once offspring disperse they do not feed again until they are adults. The speed with which they can disperse reflects the rate at which they are able to utilize the carcass. (**b**) The mass of the dispersing broods, reared under different parental social groups. Mass of each dispersing larva was measured individually, but data were analysed as mean offspring mass to avoid inflating degrees of freedom. Once offspring disperse, they do not feed again so final adult size is determined by mass at dispersal, and larger males and females are more likely to successfully defend resources for breeding. (**c**) Offspring survival to adult, measured as the percentage of offspring that dispersed that survived to adult pupation, under different parental social groups. Data are presented using box plots, with medians (inner line; where there is no inner line, the median and 25% quartile overlap), 25 and 75% quartiles (boxes) and whiskers (end of box to 1.5 × interquartile range). The dots are values that fall outside the interquartile range. Analyses are based on 138 biparental families, 119 uniparental female families and 13 uniparental male families.

We allowed individuals the freedom to adopt any of the different forms of care without experimenter interference (see Methods) so that we could measure the success of each form of care. The form of the social condition did not influence offspring performance or fitness. Both female-only and biparental family social conditions were common. Of the 269 pairs, in 138 pairs the male abandoned (51%) to leave a uniparental female, and 119 pairs (44%) remained biparental. The female abandoned in 13 cases (5%) resulting in uniparental males. The duration a parent remained with the family depended on the family social condition and the sex of the parent (analysis of variance (ANOVA), biparental female *N*=119, uniparental female *N*=138, biparental male *N*=119; uniparental male *N*=13; *F*_3,385_=86.820, *P*<0.0001). This difference was driven entirely by males who remained with females (biparental males), who abandoned the family at around 60 h (Fig. 2). Duration of attendance by biparental males was significantly less than others (*P*<0.0001). Moreover, there were more extreme values (that is, which fell outside of the interquartile range) in the amount of time a male remained in a biparental family before dispersing (Fig. 2). There was no significant difference between the duration of attendance by biparental females and uniparental females, uniparental males (Tukey–Kramer honest significant difference (HSD) *post hoc* comparison of pairs of treatments, all *P*>0.8) as all three remained with the offspring for around 100 h (Fig. 2). The family social condition (biparental *N*=119, uniparental female *N*=138, uniparental male *N*=13) had no significant effect on the duration of offspring development on a carcass (ANOVA; Fig. 3a; *F*_2,267_=1.050, *P*=0.352), their mass at dispersal (ANOVA; Fig. 3b; *F*_2,267_=0.436, *P*=0.647), or their survival until adulthood (ANOVA; Fig. 3c; *F*_2,267_=0.864, *P*=0.423).

### Transcriptomics during parenting

We next experimentally enforced different forms of care to observe changes in gene expression. Across all of the parenting versus control comparisons, we found 867 unique genes to be differentially expressed in at least one of the contrasts. These defined a set of genes we call the ‘caring gene set’ (see Methods and Supplementary Data 1). Comparing parents versus controls, the number of differentially expressed genes varied greatly (Table 1). Overall, females showed more differentially expressed genes during parenting than males, and uniparental treatments showed more differentially expressed genes than biparental treatments. Although the number of differentially expressed genes was greater for both males and females in uniparental treatments, this increase is proportionally much greater for males (Table 1; Supplementary Fig. 1). Few of the caring gene set remained differentially expressed in post-parenting versus control comparisons (Table 1; Supplementary Figs 2,3) showing that the majority of these genes return to control levels once caring has ended, strongly supporting the interpretation that differential expression of these genes contributes to a change in behavioural state between parenting and non-parenting. It is possible that differential detection of genes in males and females due to sex-biased expression could influence our results, however, we found that very few genes in our reference transcriptome assembly were only detected in one sex (46 in males and 23 in females) and none of these genes were differentially expressed between any of our treatments.

**Figure 4:**
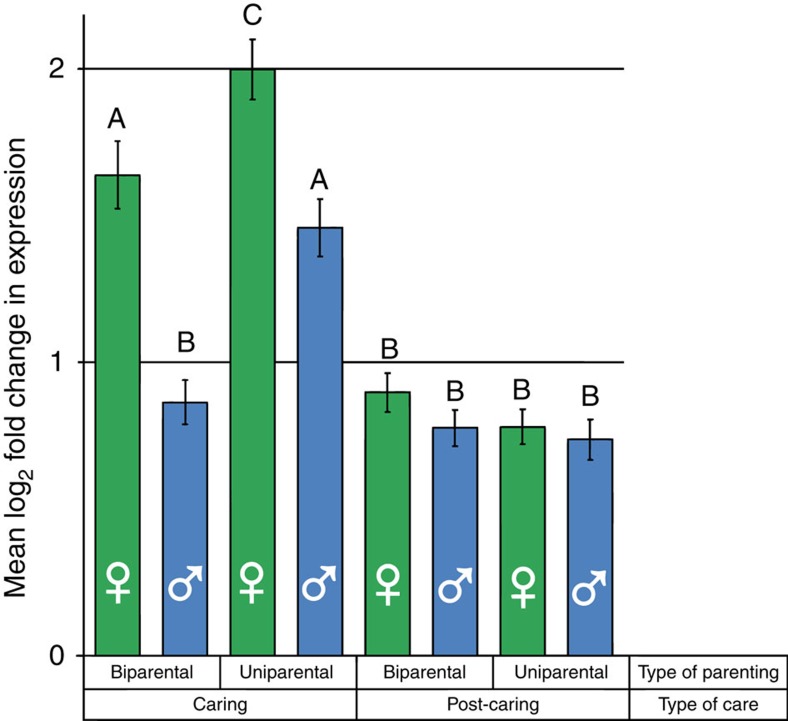
Extent of differential expression of the caring gene set under different family social conditions, measured by log-fold difference in expression. Groups with significantly different (Tukey’s HSD *P*<0.05) change in gene expression are marked with different letters. Analyses are based on two biological replicates per treatment.

The extent of the change in gene expression in the caring gene set was significantly higher for females during caring, especially when uniparental (Fig. 4, Table 2). In addition, we found significant interactions between sex and parental type, sex and caring state, and parental type and caring state (Table 2). This is because biparental females, uniparental females and uniparental males, all showed a larger change in gene expression in caring comparisons than in post-caring comparisons (Tukey’s HSD *P*<0.00001), whereas biparental males did not (Tukey’s HSD *P*>0.4). In addition, although there were greater changes in gene expression for males and females parenting when uniparental than when biparental, the difference was smaller for females than for males (female increase=22%, male increase=69%, Fisher’s exact test *P*=6.96 × 10^−10^).

**Figure 5:**
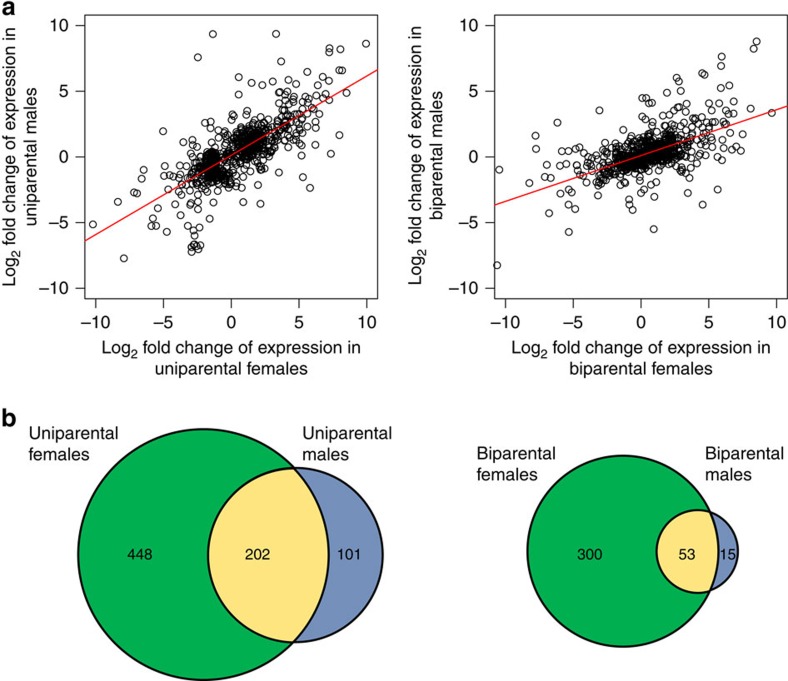
Correlation between gene expression of males and females under different family social conditions. (**a**) Correlation of male and female gene expression change in caring versus control comparisons in the caring gene set in uniparental and biparental conditions. (**b**) The number of differentially expressed (DE) genes for males and females in caring versus control comparisons in the caring gene set and the number shared in uniparental or biparental conditions (yellow areas). Note that only genes that were DE in the same direction were included in the region of overlap.

**Figure 6:**
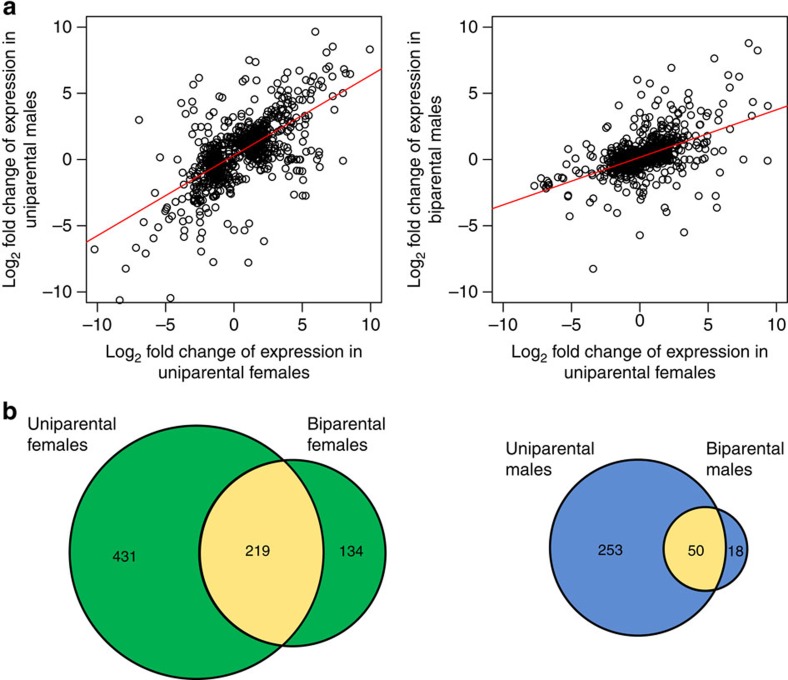
Correlation between gene expression under uniparental and biparental social conditions for each sex. (**a**) Correlation of expression change in caring versus control comparisons in the caring gene set for females and for males in uniparental and biparental conditions. (**b**) The number of differentially expressed (DE) genes in caring versus control comparisons for uniparental and biparental treatments and the number shared by females and males (yellow areas). Note that only genes that were DE in the same direction were included in the region of overlap.

Although there were unique differentially expressed genes associated with parenting in uniparental females, uniparental males, biparental females and biparental males (Supplementary Fig. 1; hypergeometric tests, *P*<0.026), there was also substantial overlap and correlation of gene expression between groups. Male and female gene expression changes in the caring gene set were correlated more strongly in uniparental than biparental comparisons (uniparental *r*=0.74, biparental *r*=0.58, Fisher’s *Z*=5.71, *P*<0.001; Fig. 5a). In addition, the slope for uniparental comparisons is around twice that of the biparental comparisons (Fig. 5a; uniparental=0.61, biparental=0.36), in agreement with the gene expression changes described above. Males and females in uniparental comparisons also shared more differentially expressed genes than biparental comparisons (Fig. 5b). Gene expression changes between uniparental and biparental treatments were also more strongly correlated for females than males (female *r*=0.65, male *r*=0.53, Fisher’s *Z*=3.93, *P*<0.001; Fig. 6a) and females shared more differentially expressed genes between parental states than males (Fig. 6b; for additional comparisons, see Supplementary Figs 1–3).

In the above analysis, we excluded contigs that did not produce a significant blast hit to an arthropod. As this may bias differentially expressed downward, we repeated the analysis but included contigs that produced a significant blast hit to an arthropod (17,019) and contigs that did not produce a significant blast hit (25,216). This expanded analysis produced remarkably similar results (Supplementary Table 1). The number of differentially expressed genes in each of the conditions is similar to the main analysis in the manuscript (Table 1, Supplementary Table 1), and the patterns of gene expression also remained qualitatively the same (Table 2; Figs 4, 5, 6; Supplementary Table 2; Supplementary Figs 4–6). Once again, uniparental males and females show more similar gene expression changes when parenting than do biparental males and females (uniparental *r*=0.68, biparental *r*=0.57, Fisher’s *Z*=5.476, *P*=4.24 × 10^−8^).

**Figure 7:**
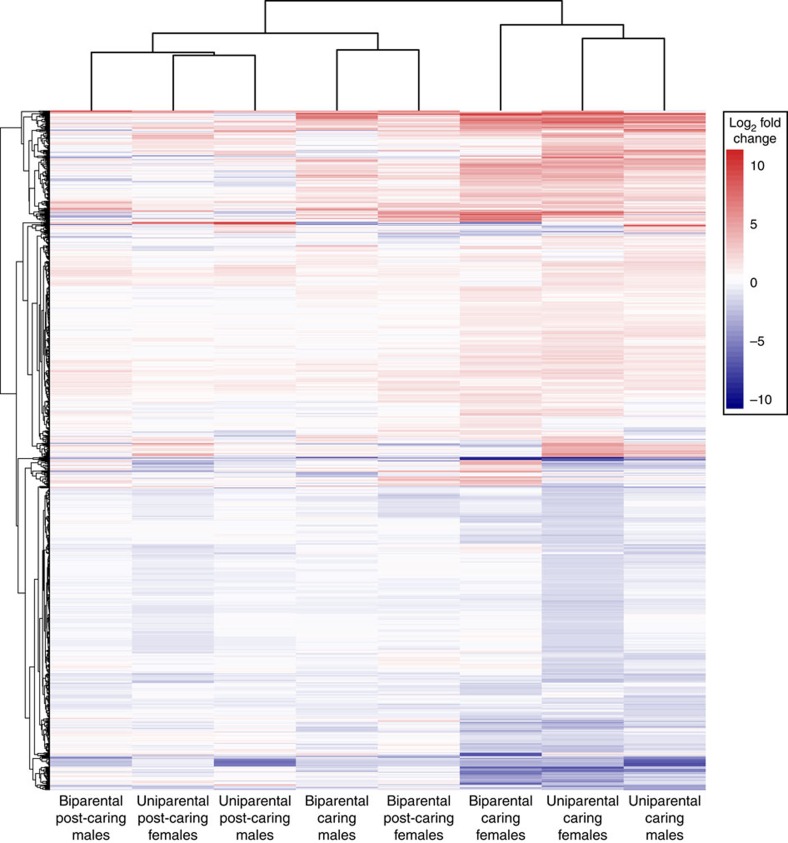
Gene expression changes in different family social conditions. A heat map of gene expression change from control samples in the caring gene set for males and females under uniparental and biparental social conditions. The dendrogram on the *x* axis shows which samples are most similar to each other. The dendrogram on the *y* axis shows how genes in the caring gene set were clustered by average fold change across the samples. Increases in gene expression from control samples are shown in red and decreases are shown in blue.

Overall, we found that when with offspring, uniparental males and females show similar changes in gene expression, while gene expression in biparental males and females was much less alike (Fig. 7). This does not appear to reflect behavioural specialization by males and females in biparental families; rather, it appears to primarily reflect a reduced transcriptional response in biparental males whereby males do not differentially express most of the genes in the caring gene set when a partner is present. Males are capable of expressing such genes, as uniparental caring males show gene expression changes similar to caring females, but they do not do so in biparental families. Female gene expression is similar under both uniparental and biparental conditions.

### Candidate genes

The caring gene set showed a significant overrepresentation of several Gene Ontology (GO) terms (Supplementary Table 3). GO terms provide information on previously implicated functions for a gene. By associating GO terms with genes that are differentially expressed during parenting, we can infer the basic functional processes that may be involved in parenting. Broadly, we found significant enrichment for GO terms involved with the breakdown and release of food (for example, serine-type endopeptidase activity, fatty-acyl-CoA reductase activity, oxidation–reduction process). Differential expression of such genes is consistent with the need to process several components of food when provisioning offspring or preparing the carcass for the offspring, including producing anti-microbial and anti-bacterial secretions.

We next examined the putative orthologs of genes differentially expressed when caring across all treatments (male and female, uniparental and biparental; Supplementary Data 1). As suggested by the GO term analysis, we found many genes associated with the breakdown of food (Supplementary Table 3). In particular, we found differentially expressed in several serine proteases, cytochrome P450 genes, and genes associated with fatty-acyl-CoA activity. In addition, we found upregulation of genes that may aid the preparation and maintenance of the carcass to be used as food by developing offspring. In particular, two genes associated with the breakdown of bacterial cell walls were differentially expressed: a peptidoglycan recognition protein (*pgrp*) and a lysozyme were both upregulated when caring. We also found a gene (*pathogenesis-related protein 5*) that encodes for a thaumatin, an antifungal peptide^27^, was upregulated when caring. We verified the differential expression of genes in each of these categories: β-glucosidase, serine protease, *pgrp* and thaumatin using quantitative PCR (qPCR). All were strongly upregulated during caring, with no significant differences in expression in the pre- and post-caring states (Supplementary Figs 7–10). The qPCR data are available from: http://dx.doi.org/10.5061/dryad.3530j; DOI: doi:10.5061/dryad.3530j.

Two genes that were highly differentially expressed stood out as potentially playing a role in influencing the behavioural change from non-caring to caring states: *vitellogenin* and *takeout*. Both may be related to social behaviour evolution. *Vitellogenin* is associated with caste-specific behaviour in honey bees^28^,^29^, ants^30^,^31^,^32^, and parenting in burying beetles^33^. *Takeout* has been shown to be involved in circadian-influenced regulation of feeding behaviour^34^,^35^ and in sex-specific courtship^36^ in *Drosophila melanogaster*. The qPCR of an independent sample of beetles confirmed that *vitellogenin* and *takeout* were downregulated during parenting in both males and females, compared with higher expression levels seen pre- and post-caring (Supplementary Figs 11,12).

## Discussion

We explored consequences for the offspring of natural variation in parenting strategies and quantified changes in gene expression associated with transitions between different family social groups in *N. vespilloides*. Previous researchers have suggested that, although the benefits of having both parents present in *N. vespilloides* are not obvious^22^,^23^,^24^,^25^, there could be subtle differences in how the sexes parent when they are acting alone or together^22^,^37^. Here, even with large sample sizes and allowing individuals to adopt their family social organization (biparental or uniparental) rather than enforcing these conditions, we found that males in the biparental families appear to provide less care than females or uniparental males as we did not find any reduction in offspring fitness associated with fewer parents. Do males adopt a different caring strategy when associating biparentally, or simply reduce the total level of care given? To address this, we used a separate experiment where we looked at patterns of gene expression and found that uniparental males and females, and biparental females, show more similar gene expression changes when parenting than do biparental males. However, rather than this suggesting specialization of parenting roles, we found that the reduced similarity of gene expression in males associating biparentally largely results from an overall lack of differential expression, rather than transcription of alternate gene sets. Uniparental and biparental females also show differences in gene expression, but these are much smaller than those between uniparental and biparental males. Hence biparental females may also behave somewhat differently, but phenotypic and genetic data together suggest that any such differences are much smaller than those seen in males. Taken together, our results suggest that there is little selection for or against any particular form of care, as long as sufficient care is provided, and that the differences we see reflect proximate differences in gene expression in males. Previous behavioural studies have found that males and females in biparental social conditions tend to behave differently, even when the study focuses on males that actively provide at least some care^22^,^23^,^25^. Both males and females can be equally effective parents, so males associating biparentally may simply persist primarily as a fail-safe against losing the female parent. ‘Biparental’ in *N. vespilloides* therefore describes the number of parents present rather than the number of parents providing direct care.

By examining the gene expression changes associated with a parenting strategy, we also uncover some of the potential proximate mechanisms involved in care. There is now extensive evidence that changes in behavioural states are associated with large scale changes in transcriptomics^18^,^19^,^38^. Parenting, both in general^17^ and in *N. vespilloides* in particular^39^, is a complex trait. In *N. vespilloides*, parental care comprises provisioning of offspring (direct care), and maintenance and guarding of the carcass and brood (indirect care). Elements of indirect care occur both before and after the offspring arrive, while direct care involving guarding or feeding of young only occurs when there can be interactions with the offspring. Previous work suggested that although biparental males provide less direct care they might provide more indirect care^37^. Our genetic evidence provides little support for such subtle specialization. We found that several antifungal and antibacterial genes, which are presumably involved in carcass maintenance, were upregulated during parenting in biparental males. However, most of these are also upregulated to a similar level in biparental females, along with several additional related genes. Another potential area for male parental specialization is guarding behaviour^40^. Changes in aggression and resource defence are known to involve extensive gene expression changes in other taxa^41^,^42^,^43^. Thus, if biparental males were specializing in different tasks associated with parenting (such as guarding), we might expect this to be reflected in more pronounced expression changes in an alternate set of genes from biparental females, rather than overall fewer changes in expression of the genes associated with the ‘caring gene set’.

Direct parental care in *N. vespilloides* involves the breakdown and provision of food^22^,^37^. Our analysis of enriched GO terms of genes differentially expressed during parenting shows an overrepresentation of several metabolic GO terms (Supplementary Table 3). The finding of such genes may be a little surprising as we targeted brain tissue for our extractions; however, our samples also contain fat bodies and connective tissues from the head. We also find upregulation of several serine proteases, the enzymes involved in digestion of dietary proteins and immune function and several genes belonging to the cytochrome P450 protein family, which contains a diverse range of enzymes. Intriguingly, we also find differential expression of several genes in the fatty-acid synthesis and fatty-acyl–CoA pathways, which have been linked to motivation of feeding behaviour in mice^44^,^45^, though there is currently no known link to a behavioural phenotype in insects. In addition, we find evidence for upregulation of several fungicidal and antibacterial genes (Supplementary Figs 8–10).

Some of the genes that we found to be differentially expressed during parenting have been implicated in related behaviours in other species. We found that orthologs of *vitellogenin* (a yolk egg protein) and *takeout* (a juvenile hormone-binding protein associated with feeding and courtship) were differentially expressed in both males and females during caring, and verified differential expression of these genes in both males and females with quantitative real-time PCR (qRT–PCR; Supplementary Figs 11,12). The *vitellogenin* and juvenile hormone have previously been linked to social behaviour in social insects castes^28^,^29^,^46^,^47^,^48^,^49^, and in a separate study, we have shown that *N. vespilloides vg* is assocatied with transitions to parenting in both males and females^33^. The ‘reproductive ground plan hypothesis’ proposes that during the evolution of eusociality, *vitellogenin* and juvenile hormone were co-opted from their original roles in regulating reproduction, into a new role of regulating social behaviour^28^,^29^,^46^,^47^,^48^,^49^. It is not clear at what stage during the evolution of eusociality these genes were co-opted into such a role, but the involvement of *vg* here suggests that these genes may have a general role in the evolution of social behaviour. The *takeout* has been shown to be involved in both temporal and satiation-associated regulation of feeding behaviour in *Drosophila melanogaster*^34^,^35^ and male courtship^36^. The relationship of *takeout* to circadian rhythms is intriguing because timing genes have been hypothesized to influence the shift to parental care in *N. vespilloides* given this is sensitive to light pulses^50^. *Takeout* is also a member of the haemolymph juvenile hormone-binding protein family of proteins, responsible for the transport of juvenile hormone from its synthesis in the corpora allata to target tissues, by preventing its breakdown in the haemolymph by nonspecific esterases^51^,^52^. Previous work in the related *N. orbicollis* has shown that juvenile hormone levels are high during parenting in both males and females^53^ and thus changes to the expression of *takeout* could possibly mediate such changes in juvenile hormone level.

Overall, our results suggest that males and females are equally effective parents alone or together, that there are identifiable changes in gene expression associated with active parenting of offspring, that biparental males provide reduced care whereas females do not and that there is no evidence for specialization of parental role. Thus, the family social condition and the number of active parents may not overlap. The lack of differences in fitness between biparental care and uniparental care suggests a plastic response by males to the presence of a caring female. This flexibility in male behaviour will cause conflict between the sexes during biparental care. This likely conflict between the sexes raises the question of why parents do not desert their partners more often. Females may even benefit by male desertion^54^. A partial explanation may be that there are limited opportunities for additional broods available to parents that desert, because breeding opportunities for *N. vespilloides* are typically limited by the availability of vertebrate carcasses^20^. Additional breeding opportunities are therefore unpredictable due to the need for this stochastically available resource. It may be more beneficial for males to remain with females to act as a parental assurance plan, taking over parenting if the female is lost^40^. This situation provides a striking contrast to one of the other few systems with flexibility in which parents care. In the Eurasian penduline tit (*Remiz pendulinus*), males and females can also both raise offspring equally competently but 30–40% of nests end up being deserted by both parents and no biparental pairs have ever been observed^55^. The difference in desertion rate may be owing to relatively unlimited breeding resources and multiple broods in a breeding season^13^,^56^. Regardless of the causes of staying or leaving the offspring, our work suggests that in *N. vespilloides*, biparental and uniparental refers to the family social organization rather than to the form of parenting that is experienced by offspring and this should result in strong sexual conflict. Further studies are needed to assess the extent to which social condition defines functional aspects of behaviour, and the extent to which there are molecular signatures of sexual conflict.

## Methods

### Behavioural methods

We first examined the effects of different social conditions (uniparental male or female and biparental) on offspring survival and performance. To establish our treatments, in the fitness study we allowed males and females to determine the social condition that they occupied. Most studies of burying beetle parenting have used laboratory setups with no opportunity for desertion^22^,^37^. In these setups, males and females are randomly paired in a container with a lid and no opportunity to desert. Here we set up 269 sexually mature pairs on individual carcasses in 17 × 11 × 6 cm clear plastic boxes without a lid and checked every 12 h for parenting by one or both of the pair. Individuals were considered to have abandoned their offspring when they were found away from the carcass and offspring for three consecutive observations^57^. Beetles rarely deserted by flying away, but instead moved as far from the carcass as possible and buried themselves in the soil.

Care is defined as both indirect and direct care^39^. Indirect care is preparation and maintenance of the carcass used to feed and as food for offspring. Direct care is the preparation of pre-digested food and regurgitation of food directly to begging offspring. Reproduction in burying beetles occurs on vertebrate carcasses, which are used as food for developing offspring. Females and males initially prepare a carcass by stripping the fur (feathers or scales, depending on species) and partially digesting the skinned carcass so that it forms a ball in a shallow depression. The parent then chews a shallow opening to which the newly hatched larvae arrive and beg. Both direct and indirect care are easily observed, unambiguous and scored as the number of occurrences in a defined timeframe^14^,^15^,^16^,^17^,^32^,^33^.

Fitness consequences of care were measured by counting the number of offspring produced in a brood, and by measuring offspring performance as development and growth. The size of the brood is under parental control^39^. The mass of the offspring and the rate at which they develop are strongly influenced by parenting and related to fitness^37^,^39^,^57^,^58^,^59^,^60^. Family means are analysed here to avoid over-inflating degrees of freedom given the unit of interest is the parent rather than individual offspring. See refs 33, 57, 58 for details on life history, the origins of the beetles used, maintenance of stock cultures and more detailed methods for measuring fitness and life-history traits.

### Transcriptomics

We generated three treatment groups; *control* (mated but no further social interactions), *parenting* and *post-parenting*, which reflect sequential social and behavioural states during a natural breeding cycle, to look for differentially expressed transcripts underlying active parenting. In this experiment, the experimenter determined the social group. We used separate control (and post-parenting) groups for both the uniparental and biparental treatments giving a total of six experimental treatments for the four social conditions, each replicated twice (Supplementary Fig. 13). For logistic reasons, we used only one pre-caring developmental stage, mated but not yet on a resource that stimulates breeding; other research examining expression changes of single genes confirms this is a valid control^33^,^61^. In this second experiment, unrelated females and males were placed into plastic mating boxes (17 × 12 × 6 cm) filled with 2.5 cm of moist soil. The control group had extended social encounters and opportunities to mate (which occurs repeatedly regardless of the presence of a resource^59^) but no opportunity to reproduce, because they were not provided with a mouse carcass. We treated the parenting group the same, but also provided a 20–24 g previously frozen mouse carcass. These individuals prepared the mouse and produced offspring. To generate uniparental treatments, we removed one mate at 60 h post pairing to leave the remaining mate in a uniparental condition. In biparental treatments, only pairs where both parents remained on the carcass throughout the caring period were used. The biparental males were collected only if they were observed near the young, so that parenting was feasible. Individuals in any of the parenting conditions were only collected when they were on the mouse near the larvae. We generated post-caring individuals the same way as parenting individuals, except they were collected only after they had spent at least 4 days on the carcass with the larvae, and had then deserted. Active parental care lasts for 72–96 h in *N. vespilloides*^60^, and by 120 h all parents had abandoned the larvae. All individuals were placed into individual containers after removal and the head tissue harvested 24 h later. Each pairing was monitored every 12 h until larvae arrived at the carcass (if applicable) to ensure each breeding pair was progressing as expected through the breeding cycle. All the treatment groups were reared in an incubator (22+0.1 °C) with a 15:9 light:dark cycle). Twenty individuals per treatment per sex were collected to generate two biological replicates of 10 pooled individuals per sex.

For control and parenting treatments, beetles were paired at 17 days post eclosion and then tissue was harvested at 21 days post-adult eclosion. For post-parenting beetles, the experimental treatment was longer (8 days). To ensure that individuals from all the treatments were age-matched at the point of tissue collection, beetles were paired at 13 days post eclosion to harvest tissue at 21 days post-adult eclosion.

Our samples consisted of whole heads, mainly including brain tissue but also including associated brain fat body and connective tissues. Brains and associated tissues from the head were dissected within 6 min of decapitation and placed in 50 μl of Ambion RNAlater and stored at −20 °C before RNA extraction. Tissue from whole heads from each treatment was pooled into two samples with 10 brains in each sample. RNA was extracted from each sample using the TRIzol Plus RNA Purification Kit (Life Technologies). Purity of the RNA was checked using NanoDrop ND-1000 spectrophotometer (NanoDrop Technologies) and integrity with 2100 Bioanalyzer (Agilent Technologies).

### Sequencing

Samples were sequenced at Beijing Genomics Institute (Shenzhen, China) using an Illumina HiSeq 2000 to produce a total of 713,929,590200, bp insert paired-end reads (90+90 bp). Reads were quality trimmed (bases with a phred score of <20 or two consecutive bases with a phred score <32 were trimmed) and screened for adaptor sequences using trimmomatic (v. 0.30; ref. 62). Reads containing adaptor sequence or that had a length of less than 85 after quality trimming, were discarded (along with their associated paired read). From this trimming, we discarded 4,784,135 read pairs (0.67%).

### *De novo* transcriptome assembly and annotation

Trimmed reads were assembled using SOAPdenovo-trans^63^ with default parameters (minimum contig size=300) to produce a *de novo* transcriptome assembly. To optimize k-mer length, several assemblies were produced with varying k-mer length. The optimum k-mer length was 25 as determined by several metrics: N50, the number of contigs over 1,000 bp in the assembly, number of ‘core eukaryotic genes’ present in the assembly identified using the CEGMA pipeline^64^,^65^, and the number of *Nicrophorus* ESTs (obtained from GenBank, https://www.ncbi.nlm.nih.gov/genbank/, accessed November 9 2013) that had a blast hit in the assembly (see Supplementary Fig. 14).

This assembly consisted of 48,296 scaffolds and was annotated using Blast2GO^66^. Specifically, all contigs were blasted (blastX) to the non-redundant protein sequence (nr) database. Contigs without a significant blast hit (*E*-value >0.001) were then blasted (blastN) to the non-redundant nucleotide collection (nt). From this, we classified genes into three categories: arthropod genes (contigs that obtained a significant blast hit to any arthropod (17,019, 35%), non-arthropod (contigs that obtained a significant blast hit to anything else (6,061, 13%)), and unknown (contigs that did not obtain a significant blast hit (25,216, 52%)). As we were unsure whether the contigs that did not get a hit represent genes or possible contaminants, we excluded them to produce the reference assembly for our gene expression analyses presented in the main text (for assembly statistics, see Supplementary Tables 4,5). To determine whether excluding the unknown contigs influenced our results, we also repeated all our gene expression analyses with them included (Supplementary Figs 4–6; Supplementary Tables 1,2).

Blast2GO was also used to obtain putative function information using Interpro scan and the GO terms associated with the blast hit. GO terms give information on the functional processes in which a gene has been previously implicated. By associating GO terms with genes that are differentially expressed during parenting, we can infer which functional process may be involved in parenting.

### Mapping and detection of differential expression

Bowtie2 (ref. 67) and RSEM (RNA-seq by Expectation Maximization^68^) were used to map and assign reads from each sample to the reference assembly (for mapping statistics, see Supplementary Fig. 15 and Supplementary Table 6). SOAPdenovo-trans identified several transcripts with overlapping regions. We provided this information to RSEM, to group reads mapping to such transcripts together before expression analysis. Expression analysis was performed using the Bioconductor package EdgeR (v. 3.2.4.)^69^ in R. Normalization factors for each sample were computed using the TMM method. A generalized linear model (GLM) with negative binomial distribution was then fitted to the number of counts per scaffold with the following single terms: parenting state (uni- or bi- parental), behavioural state (control, parenting or post-parenting) and sex (male or female); along with all possible interactions: parenting state × behavioural state, parenting state × sex, behavioural state × sex and behavioural state × parenting state × sex as a design matrix (Supplementary Table 7). Dispersion was estimated using the Cox-Reid profile-adjusted likelihood method. The design matrix was then used to perform GLM likelihood ratio tests to determine the significance of a treatment effect for each scaffold by comparing control samples to either parenting samples or post-parenting samples, producing eight contrasts in all (uniparental male control: uniparental male parenting, uniparental male control: uniparental male post-parenting, biparental male control: biparental male parenting, biparental male control: biparental male post-parenting, uniparental female control: uniparental female parenting, uniparental female control: uniparental female post-parenting, biparental female control: biparental female parenting and biparental female control: biparental female post-parenting). The *P* values from the GLM likelihood ratio tests from each contrast were then individually corrected for multiple testing using Benjamini and Hochberg’s algorithm^70^ to control for false discovery rate, with statistical significance set at <5%. This identifies the scaffolds that are differentially expressed in each of the treatment contrasts with an associated blast hit. For simplicity, we refer to scaffolds as genes, and the associated blast hits as orthologs.

To quantitatively examine expression changes of genes that show differential expression during parenting, we defined a ‘caring gene set’ to contain genes found to be differentially expressed in any of the parenting versus control comparisons. To determine the effect of sex, behavioural state and parenting state on gene expression change in the caring gene set, we fit an ANOVA to the gene expression change for each gene in the caring gene set with the following terms: parenting state (uniparental or biparental), behavioural state (control, parenting or post-parenting) and sex (male or female); along with all possible interactions: parenting state × behavioural state, parenting state × sex, behavioural state × sex and behavioural state × parenting state × sex. Following this, we performed a Tukey *post hoc* pairwise comparison of means (Tukey’s HSD) to determine which groups were different from each other.

### qRT–PCR analysis

We collected whole-head samples from the same three behavioural states: mated but not yet caring for offspring, actively caring and post-caring. Both uniparental females and uniparental males were collected (see ref. 33 for an expanded version of this design and details on the definitions of the different social conditions). All individuals were 21 days post adult eclosion when collected. Heads were collected immediately into liquid nitrogen.

Total RNA was extracted from adult head samples using a Qiagen RNAeasy Lipid kit (Qiagen, Venlo, Netherlands) per manufacturer’s instructions with frozen heads homogenized in 500 μl of Qiazol in a mortar chilled with liquid nitrogen. See ref. 33 and ref. 61 for details. RNA was quantified in 1:10 dilutions using a Qubit 2.0 florometer (Invitrogen Corporation, Carlsbad, CA, USA) and cDNA was synthesized from 500 ng of RNA using Quanta Bioscience qScript reverse transcriptase master mix following manufacturer’s instructions. RNA samples were stored at −80 °C and cDNA samples were stored at −20 °C until use.

We designed qRT–PCR primers from the PCR-validated consensus sequences for each gene using Primer3 (v4.0.0). We validated primer pairs by estimating PCR efficiency and assessed the number of amplicons generated from each pair with a disassociation curve from a qRT–PCR run. We estimated PCR efficiency with a four-point, four-fold serial dilution series using a pool of common cDNA, which had been generated using the same protocol as the experimental samples. This dilution series produced a linear dynamic range encompassing the experimental variation in *C*_T_ values of all target amplicons and ensured primer pairs with efficiencies close to two.

We quantified mRNA levels by qRT–PCR on a Roche LightCycler 480 platform using Roche LightCycler 480 SYBR I Green Master Mix and a 60 °C annealing temperature. Each experiment was run on a single 364-well plate. cDNA was diluted 1:10 and 2 μl was used as input into a 10 μl reaction containing 5 μl of SYBR mix and 3 μl of a primer stock containing both sense and antisense primers at 1.33 μmol l^−1^. We ran three technical replicates and 10 biological replicates for all the treatments, and used TATA-binding protein as an endogenous control gene. This gene has previously been shown to be most stable over these social/reproductive states^33^,^61^.

We used the ΔΔ*C*_T_ method to convert raw expression data to normalized relative expression values, using the pre-caring treatment as our comparison group. The data were visually inspected for outliers. We analysed log-transformed relative expression values as these were normally distributed. We tested for the effect of social/reproductive context using an ANOVA, using JMP Pro (v10.0.1) for all statistical analyses.

## Additional information

**Accession codes:** The sequences generated in this study have been deposited in NCBI’s Gene Expression Omnibus (GEO) under bioproject number PRJNA285436. Mapped read counts have been deposited into GEO under the accession code GSE72225. Raw read have been deposited in GEO under accession codes SRR2086504, SRR2086507 to SRR2086511, SRR2089927 to SRR2089929, SRR2089932 to SR2089934, SRR2089939, SRR2089940, SRR2089944, SRR2089950, SRR2089955, SRR2089957 to SRR2089961, SRR2089992, SRR2089993. The transcriptome assembly has been deposited at DDBJ/EMBL/GenBank under the accession GDKQ00000000. The version described in this paper is the first version, GDKQ01000000.

**How to cite this article:** Parker, D. J. *et al*. Transcriptomes of parents identify parenting strategies and sexual conflict in a subsocial beetle. *Nat. Commun.* 6:8449 doi: 10.1038/ncomms9449 (2015).

## Supplementary Material

Supplementary Information Supplementary Figures 1-15, Supplementary Tables 1-7 and Supplementary References

Supplementary Data 1 Blast hits and Log2 fold change in expression for uni- and bi- parental males and females in the caring gene set

## Figures and Tables

**Figure 1:**
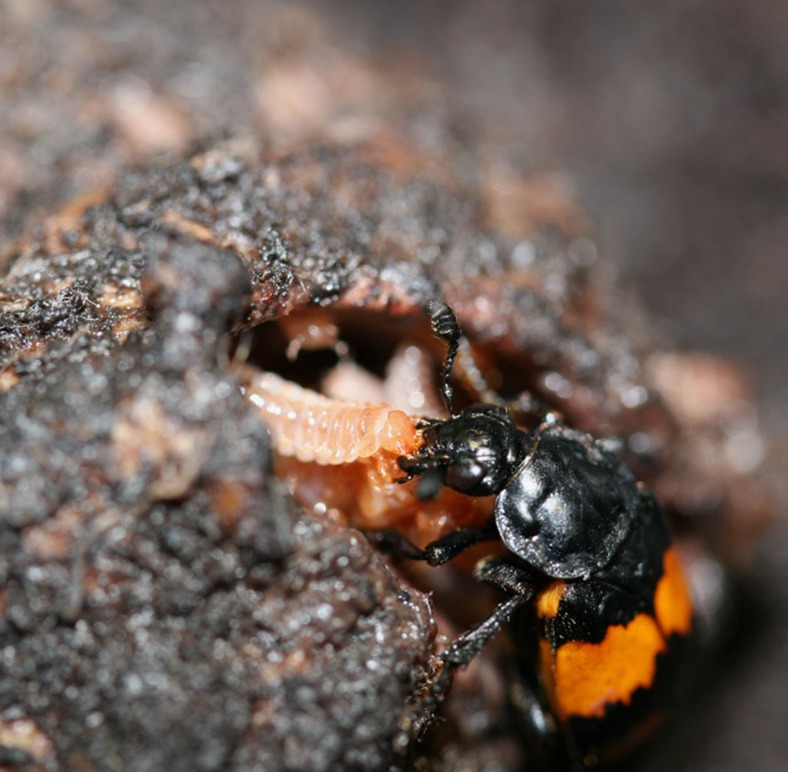
Parental care in burying beetles involves direct regurgitation of food to begging offspring. A female *Nicrophorus vespilloides* burying beetle feeds her begging young. The parent and offspring are in a mouse carcass prepared by the parent as food for the young. Photo by Allen J. Moore.

**Table 1:** The number of differentially expressed genes in the caring gene set.

**Comparison**	**Parenting type**	**Males**	**Females**
Caring versus control	Biparental	68	353
	Uniparental	303	650
Post-caring versus control	Biparental	55	53
	Uniparental	60	42

**Table 2:** The effect of sex, parental type, and caring state on gene expression change in the caring gene set assessed by ANOVA examining changes in expression of 867 genes in uniparental females, biparental females, uniparental males, and in biparental males in both caring and non-caring states.

**Variable**	**DF**	**Sum of squares**	**Mean squares**	***F***	***P*****-value**
Sex	1	236	236.4	151.67	<2 × 10^−16^
Uniparental/biparental	1	69	68.7	44.06	3.41 × 10^−11^
Caring/non-caring	1	829	829.5	532.07	<2 × 10^−16^
Sex × parenting	1	11	10.6	6.81	0.00907
Sex × caring	1	143	143.3	91.93	<2 × 10^−16^
Parenting × caring	1	133	133.3	85.52	<2 × 10^−16^
Sex × parenting × caring	1	3	2.7	1.71	0.191
Residual	6,928	10,801	1.6		
