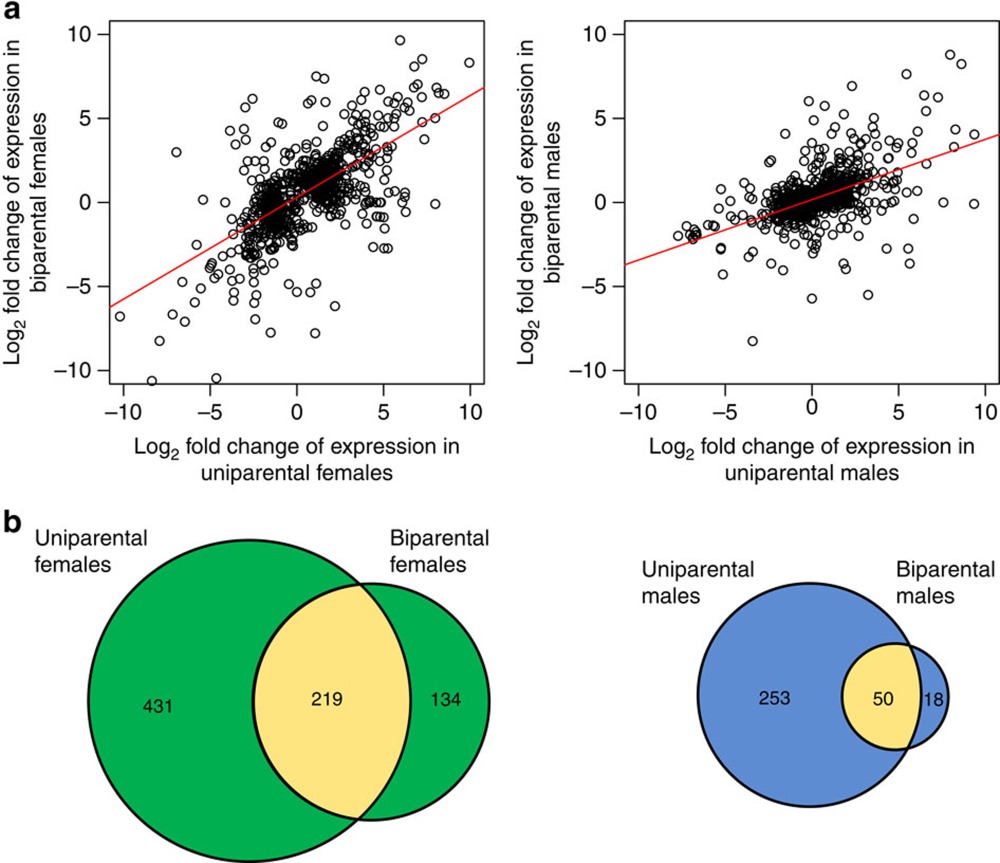# Erratum: Transcriptomes of parents identify parenting strategies and sexual conflict in a subsocial beetle

**DOI:** 10.1038/ncomms11737

**Published:** 2016-05-27

**Authors:** Darren J. Parker, Christopher B. Cunningham, Craig A. Walling, Clare E. Stamper, Megan L. Head, Eileen M. Roy-Zokan, Elizabeth C. McKinney, Michael G. Ritchie, Allen J. Moore

*Nature Communications* 6, Article number: 8449 10.1038/ncomms9449 (2015); Published: 09292015; Updated: 05272016

In the Methods section of this Article, a detail of the analysis involved in producing Fig. 4 and Table 2 was omitted. The section ‘Mapping and detection of differential expression' should state that the overall magnitude of gene expression change was examined, ignoring the direction (up or downregulation). There are also errors in the labelling of axes in Fig. 6a. The *y* axis of the graph on the left should have read ‘Log_2_-fold change of expression in biparental females' and the *x* axis of the graph on the right should have read ‘Log_2_-fold change of expression in uniparental males'. The correct version of Fig. 6 appears below.

**Figure 6:**