# Supplementary material for: Transcriptomes of parents identify parenting strategies and sexual conflict in a subsocial beetle
Source: Nat Commun. 2015 Sep 29;6:8449. doi: 10.1038/ncomms9449 (PMC4598741; doi:10.1038/ncomms9449)
Supplement: Supplementary Information — Supplementary Figures 1-15, Supplementary Tables 1-7 and Supplementary References [file ncomms9449-s1.pdf]

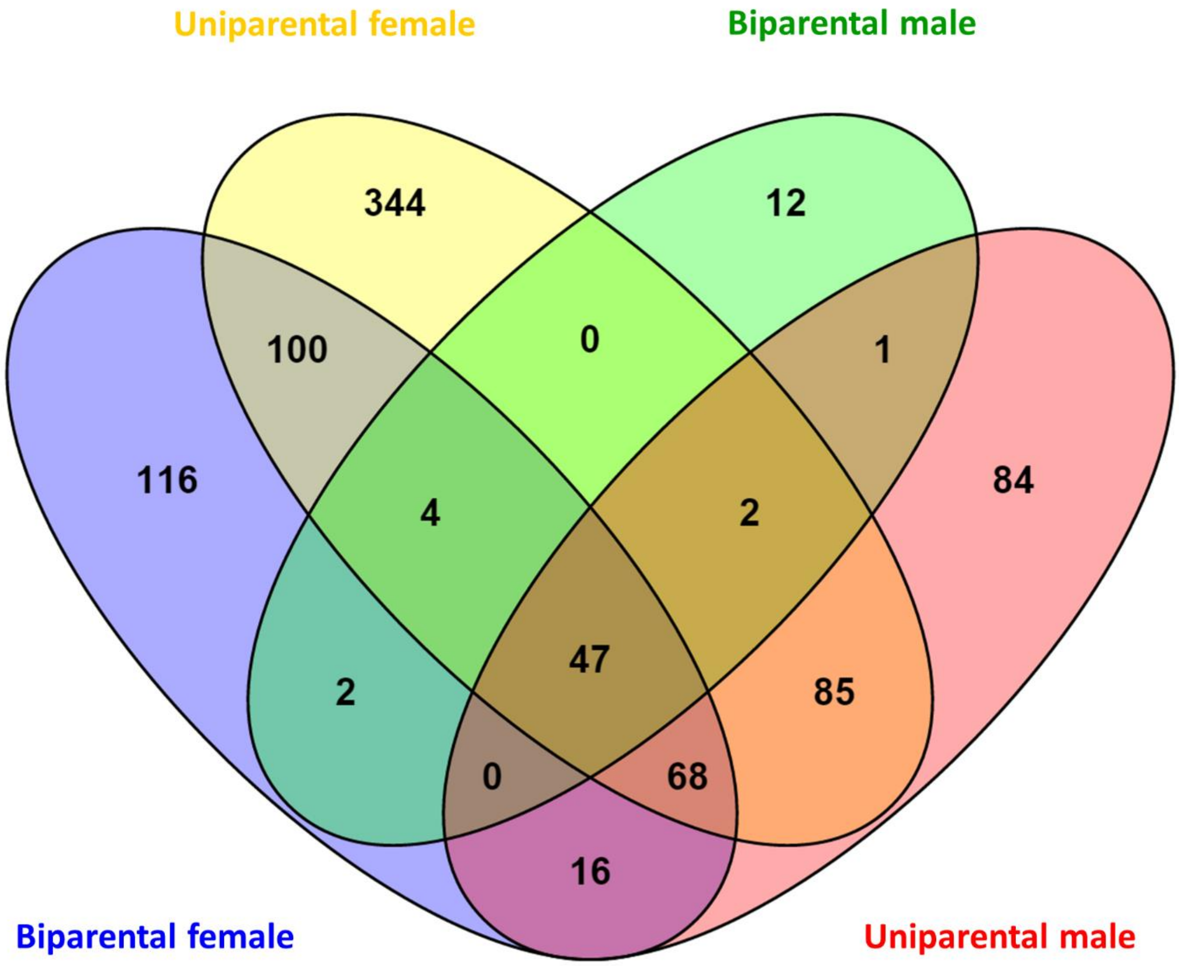

Supplementary Figure 1 | The number of differentially expressed genes for uniparental males (green), uniparental females (yellow), biparental males (red), and biparental females (blue) in caring vs. control comparisons in the caring gene set and the numbers shared between each of these (the overlap regions). Note only genes that were DE in the same direction were included in the regions of overlap.

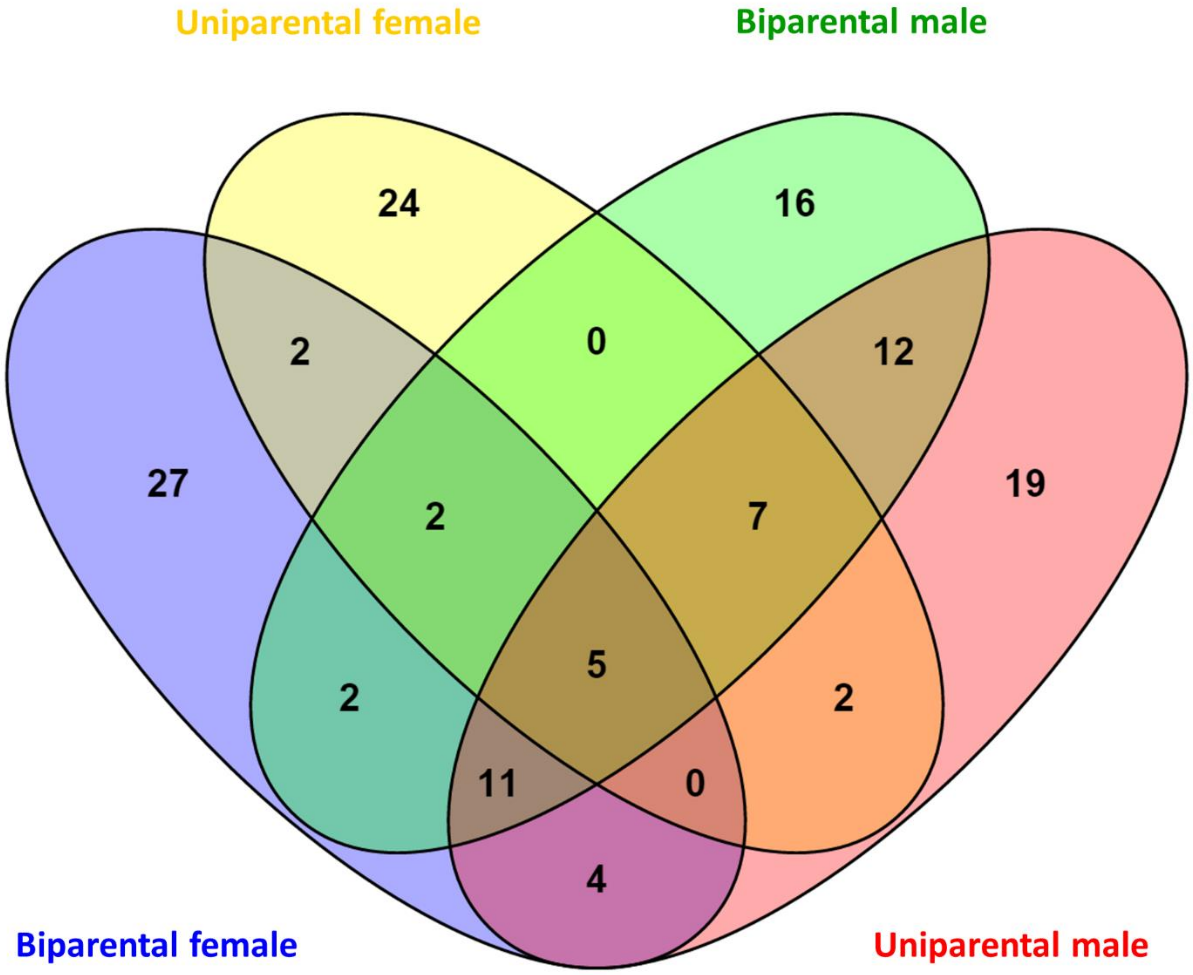

Supplementary Figure 2 | The number of differentially expressed genes for uniparental males (green), uniparental females (yellow), biparental males (red), and biparental females (blue) in post-caring vs. control comparisons in the caring gene set and the numbers shared between each of these (the overlap regions). Note only genes that were DE in the same direction were included in the regions of overlap.

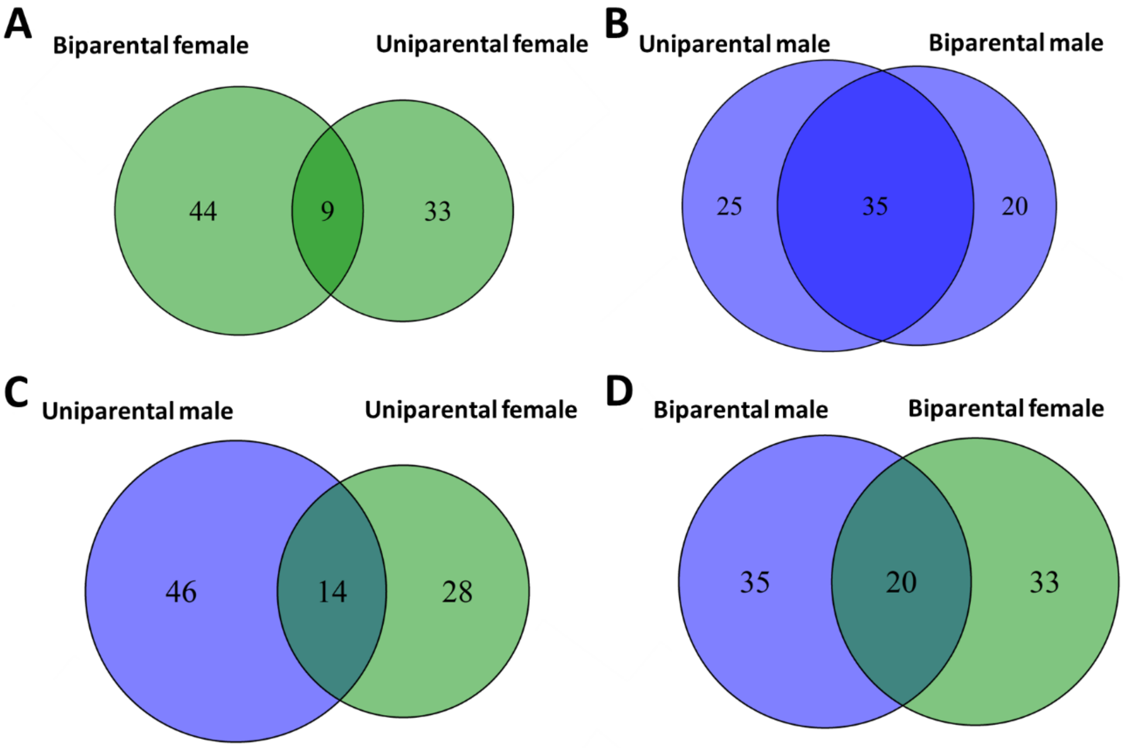

Supplementary Figure 3 | The number of differentially expressed genes in post-caring vs. control comparisons and the numbers shared (in the overlap) for: A. biparental females and uniparental females, B. uniparental males and biparental males, C. uniparental males and uniparental females, and D. biparental males and biparental females. Note only genes that were DE in the same direction were included in the regions of overlap.

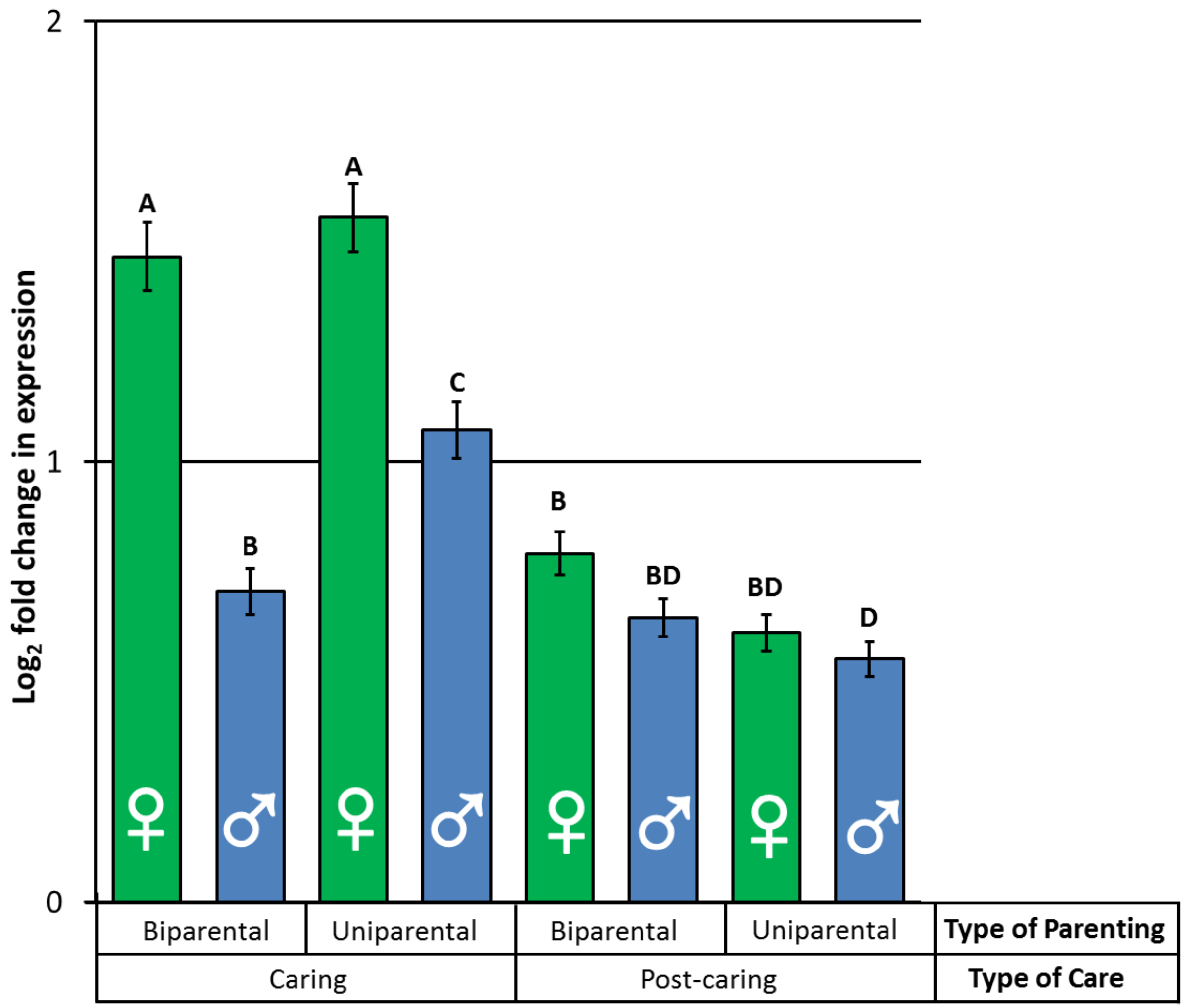

Supplementary Figure 4 | Change of gene expression in the caring gene set under different forms of parental care when using the "extended reference assembly".

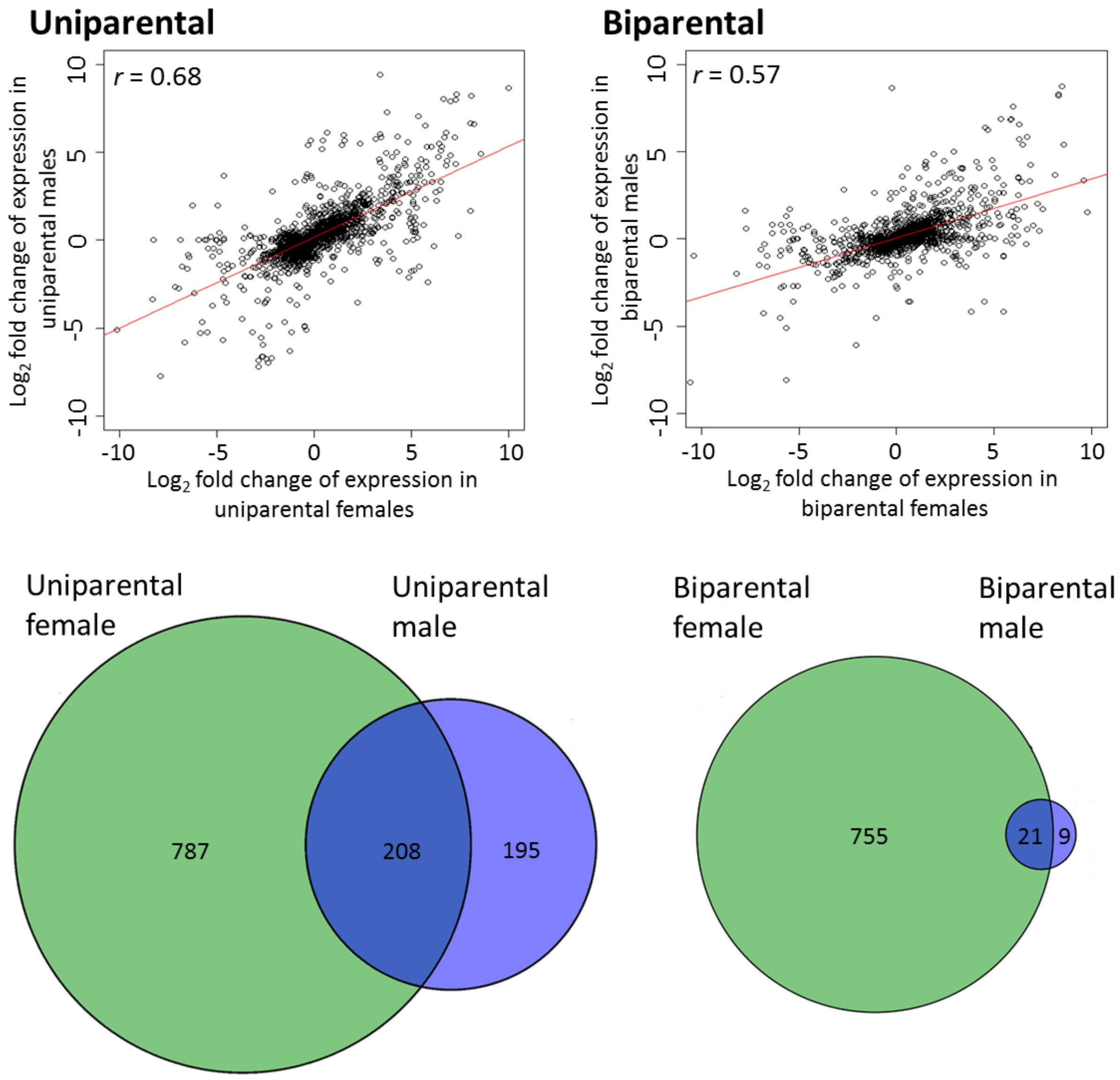

Supplementary Figure 5 | Correlation between gene expression under uniparental and biparental care when using the "extended reference assembly". (a) Correlation of expression change in caring vs. control comparisons in the caring gene set for each sex in uniparental and biparental conditions. **(b)** The number of differentially expressed genes in caring vs. control comparisons for uniparental and biparental treatments and the number shared by females and males (yellow areas).

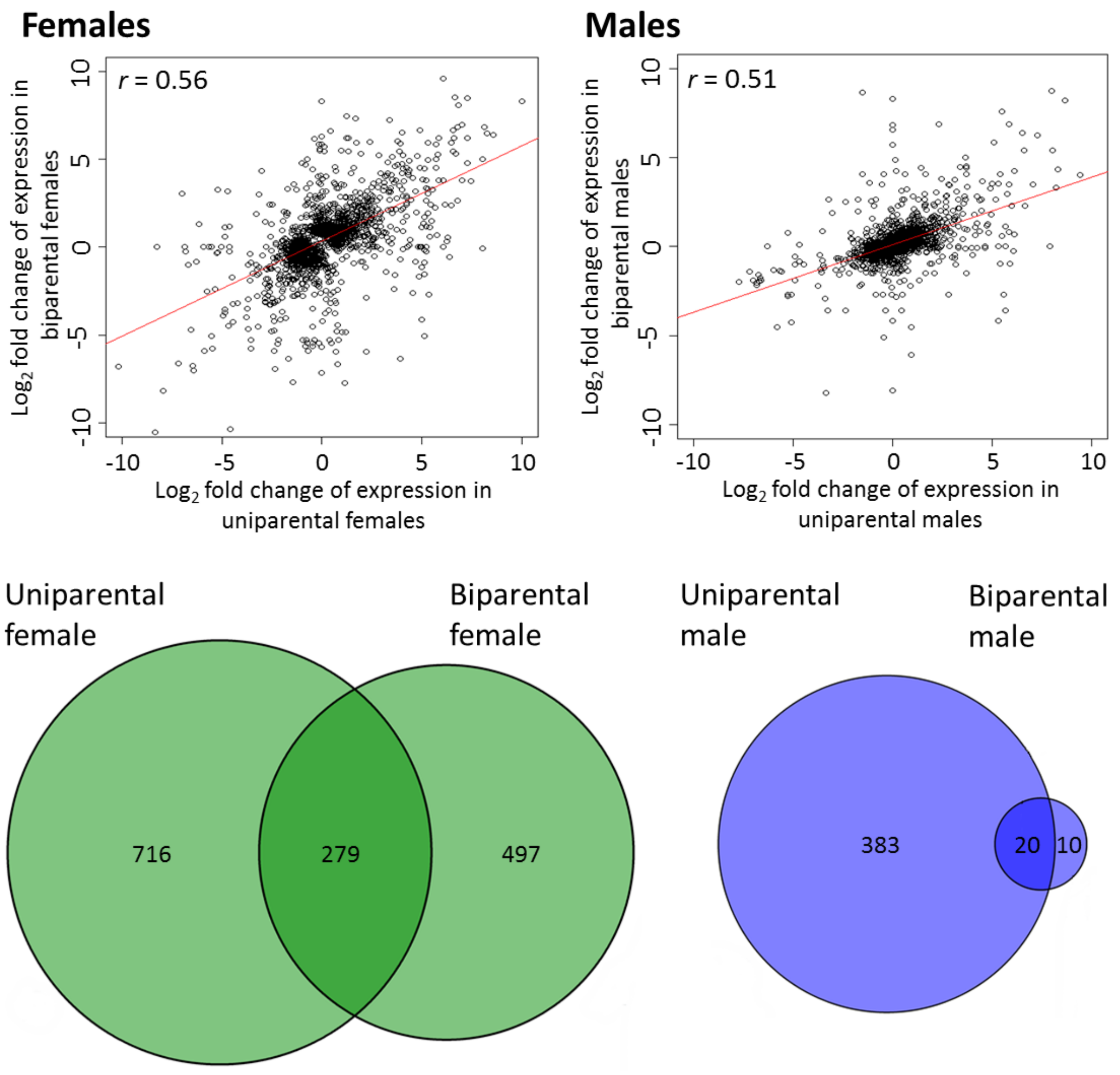

Supplementary Figure 6 | Correlation between gene expression under male and female parental care when using the "extended reference assembly". (a) Correlation of male and female gene expression change in caring vs. control comparisons in the caring gene set in uniparental and biparental conditions. **(b)** The number of differentially expressed genes for males and female in caring vs. control comparisons in the caring gene set and the number shared in uniparental or biparental conditions (yellow areas).

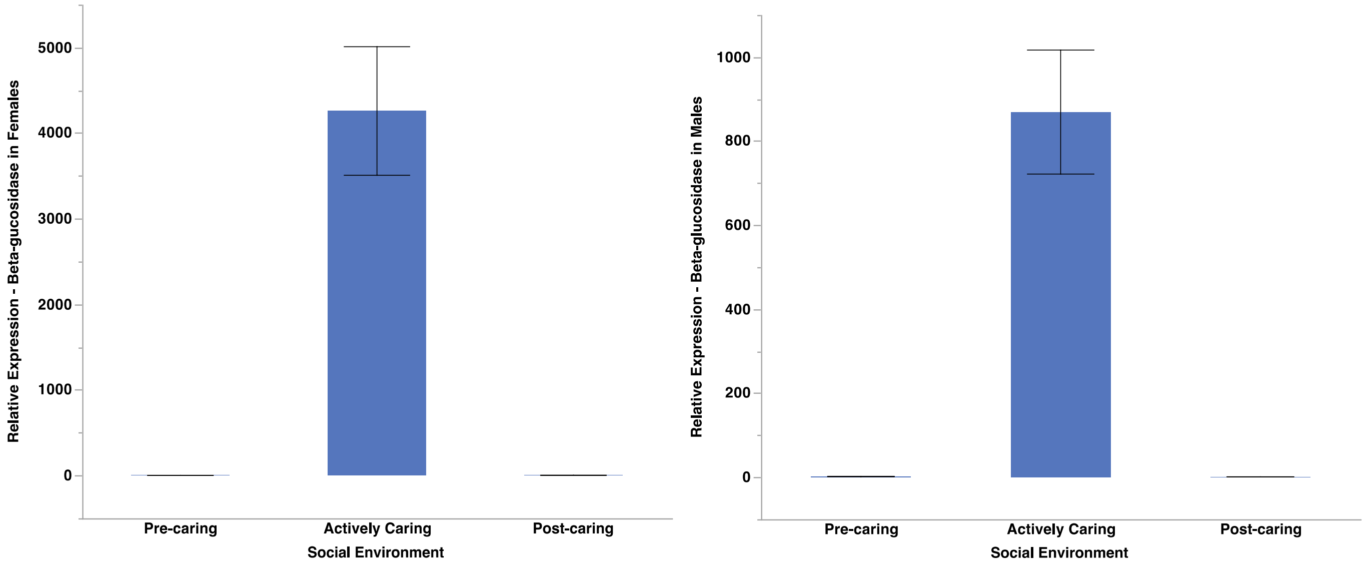

Supplementary Figure 7 | Relative expression of *β-glucosidase* under three behavioural stages – pre-caring (control, mated but not on a mouse); actively caring; post-caring (24 h after dispersal from the carcass by the caring female) in head tissue from **A**) uniparental female *Nicrophorus vespilloides* The relative expression is so much lower in pre-caring (mean = 1.56) and post-caring (mean = 2.45) states that it does not register on this graph, but it was measurable in our samples. ANOVA F=424.433, df = 2,27, P < 0.0001. Significance of pair-wise comparisons assessed by Fisher's LSD test: Pre-caring < Actively caring, P < 0.0001; Pre-caring < Post-caring, P = 0.025; Post-caring < Actively caring, P < 0.0001. **B**) uniparental male *N. vespilloides*. The relative expression is so much lower in pre-caring (mean = 1.61) and post-caring (mean = 0.95) states that it does not register on this graph, but it was measurable in our samples. ANOVA F= 310.175, df = 2,26, P < 0.0001. Significance of pair-wise comparisons assessed by Fisher's LSD test: Pre-caring < Actively caring, P < 0.0001; Pre-caring NS Post-caring, P = 0.534; Post-caring < Actively caring, P < 0.0001. In all samples, analyses performed on ln-transformed data ($-\Delta\Delta C_T$). TATA-binding protein was used as an endogenous control, with 3 technical replicates and 10 biological replicates per gene and treatment. One of the replicates failed in the pre-caring stage, so there were only 9 biological replicates. The pre-caring state was used as the baseline expression state.

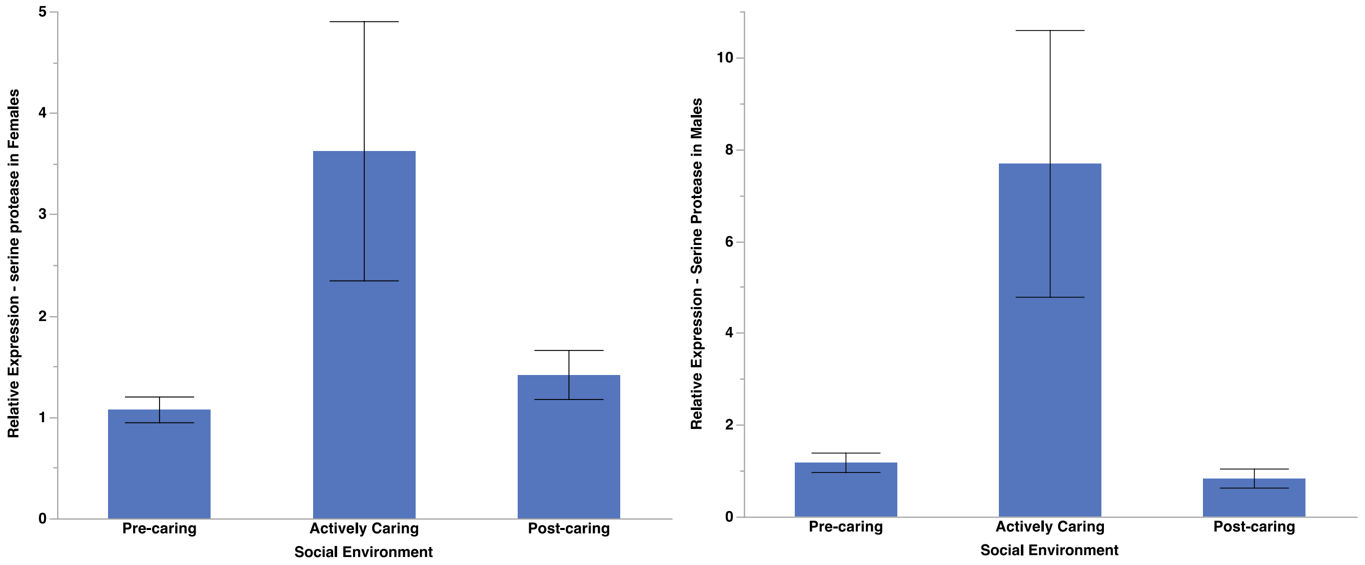

Supplementary Figure 8| Relative expression of *serine protease* under three behavioural stages – pre-caring (control, mated but not on a mouse); actively caring; post-caring (24 h after dispersal from the carcass by the caring female) in head tissue from **A)** uniparental female *Nicrophorus vespilloides*. ANOVA F= 4.119, df = 2,27, P = 0.024. Significance of pair-wise comparisons assessed by Fisher's LSD test: Pre-caring < Actively caring, P = 0.0092; Pre-caring N.S. Post-caring, P = 0.510; Post-caring < Actively caring, P = 0.042. **B)** from uniparental male *N. vespilloides*. ANOVA F= 18.152, df = 2,26, P = 0.0002. Significance of pair-wise comparisons assessed by Fisher's LSD test: Pre-caring < Actively caring, P = 0.0015; Pre-caring N.S. Post-caring, P = 0.359; Post-caring < Actively caring, P < 0.0001. Analyses performed on ln-transformed data ($-\Delta\Delta C_T$). TATA-binding protein was used as an endogenous control, with 3 technical replicates and 10 biological replicates per gene and treatment. One of the replicates failed in the pre-caring stage, so there were only 9 biological replicates. The pre-caring state was used as the baseline expression state.

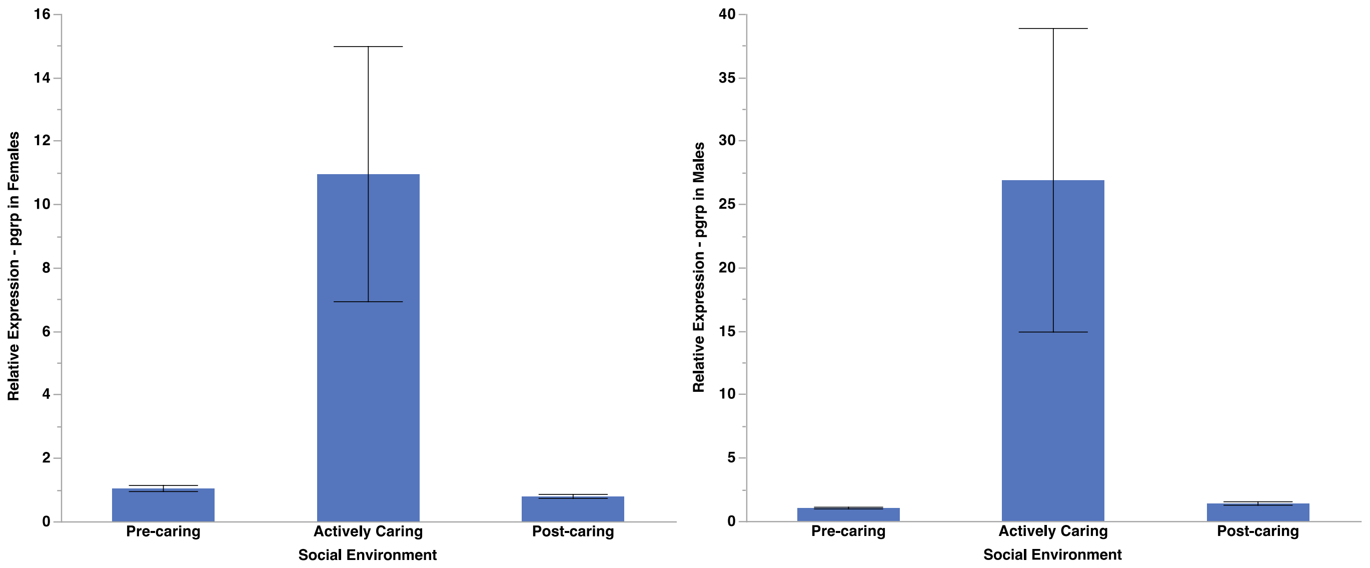

Supplementary Figure 9 | Relative expression of *peptidoglycan recognition protein* (*pgrp*) under three behavioural stages – pre-caring (control, mated but not on a mouse); actively caring; post-caring (24 h after dispersal from the carcass by the caring female) in head tissue from uniparental female *Nicrophorus vespilloides*. ANOVA F=28.350, df = 2,27, P < 0.0001. Significance of pair-wise comparisons assessed by Fisher's LSD test: Pre-caring < Actively caring, P < 0.0001; Pre-caring NS Post-caring, P = 0.388; Post-caring < Actively caring, P < 0.0001). **B)** in head tissue from uniparental male *N. vespilloides*. ANOVA F=36.790, df = 2,26, P < 0.0001. Significance of pair-wise comparisons assessed by Fisher's LSD test: Pre-caring < Actively caring, P < 0.0001; Pre-caring NS Post-caring, P = 0.518; Post-caring < Actively caring, P < 0.0001. Analyses performed on ln-transformed data ($-\Delta\Delta C_T$). TATA-binding protein was used as an endogenous control, with 3 technical replicates and 10 biological replicates per gene and treatment. One of the replicates failed in the pre-caring stage, so there were only 9 biological replicates. The pre-caring state was used as the baseline expression state.

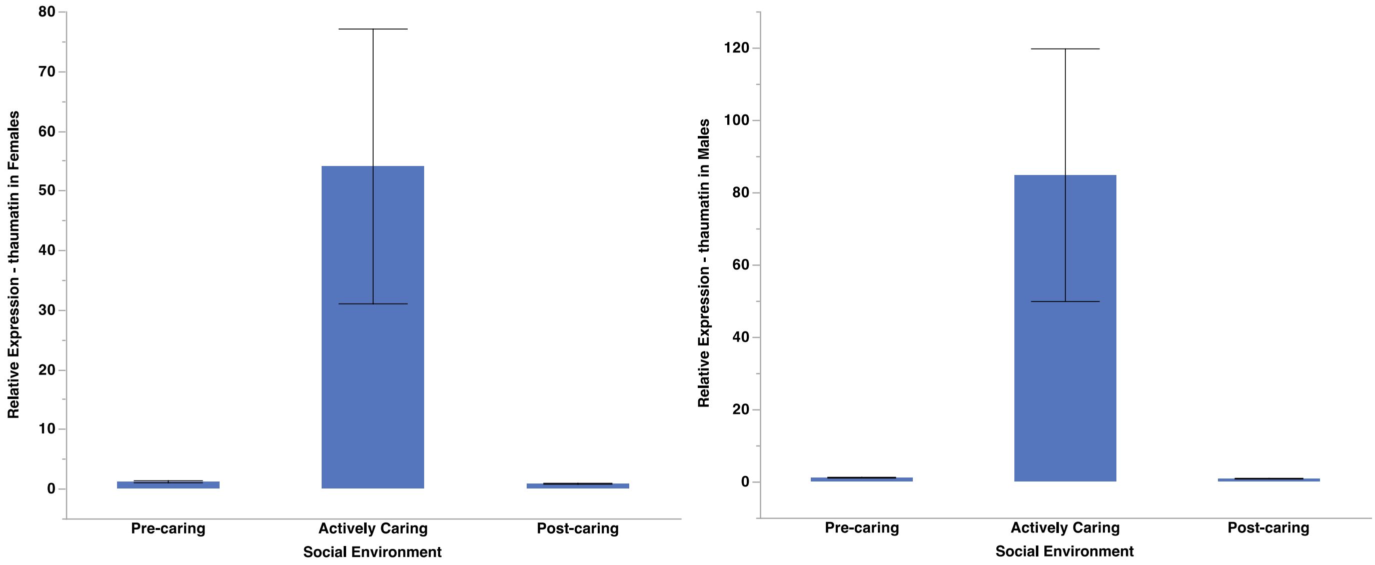

Supplementary Figure 10 | Relative expression of *thaumatin* under three behavioural stages – pre-caring (control, mated but not on a mouse); actively caring; post-caring (24 h after dispersal from the carcass by the caring female) **A)** in head tissue from uniparental female *Nicrophorus vespilloides*. ANOVA F=80.887, df = 2,26, P < 0.0001. Significance of pair-wise comparisons assessed by Fisher's LSD test: Pre-caring < Actively caring, P < 0.0001; Pre-caring NS Post-caring, P = 0.518; Post-caring < Actively caring, P < 0.0001. Analyses performed on ln-transformed data (-$\Delta\Delta C_T$). **B)** from uniparental male *Nicrophorus vespilloides*. ANOVA F=71.089, df = 2,27, P < 0.0001. Significance of pair-wise comparisons assessed by Fisher's LSD test: Pre-caring < Actively caring, P < 0.0001; Pre-caring NS Post-caring, P = 0.567; Post-caring < Actively caring, P < 0.0001. Analyses performed on ln-transformed data (-$\Delta\Delta C_T$). TATA-binding protein was used as an endogenous control, with 3 technical replicates and 10 biological replicates per gene and treatment. The pre-caring state was used as the baseline expression state.

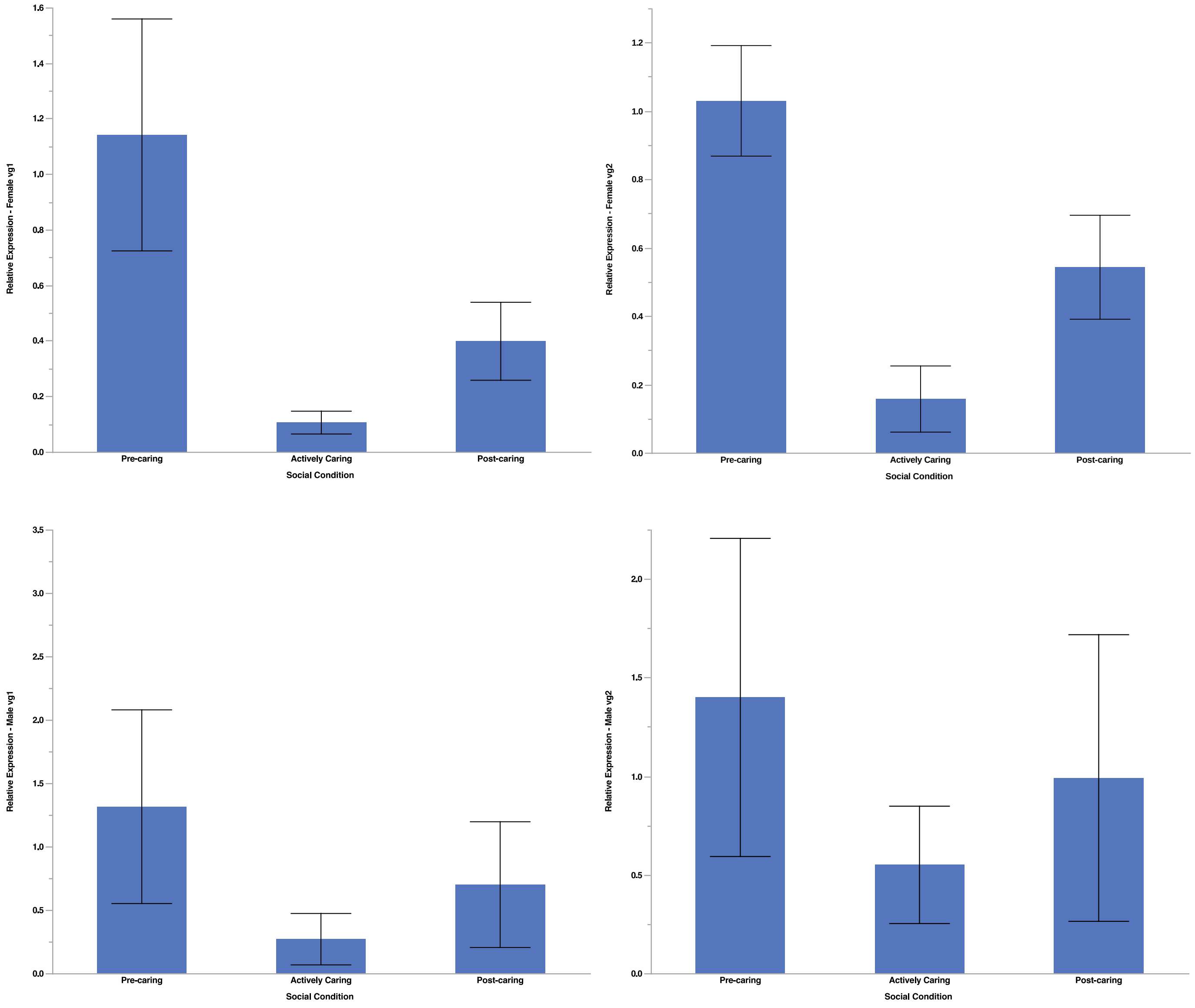

Supplementary Figure 11 | Relative expression of *vitellogenin 1* (*vg1*)[1] under three behavioural stages – pre-caring (control, mated but not on a mouse); actively caring; post-caring (24 h after dispersal from the carcass by the caring female), measured by qRT-PCR, **A)** in head tissue from uniparental female *Nicrophorus vespilloides*. Data expressed as means ± 95% CI. ANOVA F=42.719, df = 2,27, P < 0.0001. Significance of pair-wise comparisons assessed by Fisher's LSD test: Pre-caring significantly > Actively caring, P < 0.0001; Pre-caring significantly > Post-caring, P = 0.0005; Post-caring > Actively caring, P < 0.0001. **B)** in head tissue from uniparental female *Nicrophorus vespilloides*. ANOVA F=35.052, df = 2,27, P < 0.0001. Significance of pair-wise comparisons assessed by Fisher's LSD test: Pre-caring > Actively caring, P < 0.0001; Pre-caring > Post-caring, P = 0.016; Post-caring > Actively caring, P < 0.0001. Analyses performed on ln-transformed data ($-\Delta\Delta C_T$).). **C)** in head tissue from uniparental male *N. vespilloides*. ANOVA F=8.507, df = 2,27, P = 0.0014. Significance of pair-wise comparisons assessed by Fisher's LSD test: Pre-caring > Actively caring, P < 0.0003; Pre-caring NS Post-caring, P = 0.110; Post-caring > Actively caring, P = 0.021. **D)** in head tissue from uniparental male *N. vespilloides*. ANOVA F=6.192, df = 2,27, P = 0.097. Significance of pair-wise comparisons assessed by Fisher's LSD test: Pre-caring > Actively caring, P = 0.033; Pre-caring NS Post-caring, P = 0.358; Post-caring NS Actively caring, P = 0.201. Analyses performed on ln-transformed data ($-\Delta\Delta C_T$).). TATA-binding protein was used as an endogenous control, with 3 technical replicates and 10 biological replicates per gene and treatment. The pre-caring state was used as the baseline expression state.

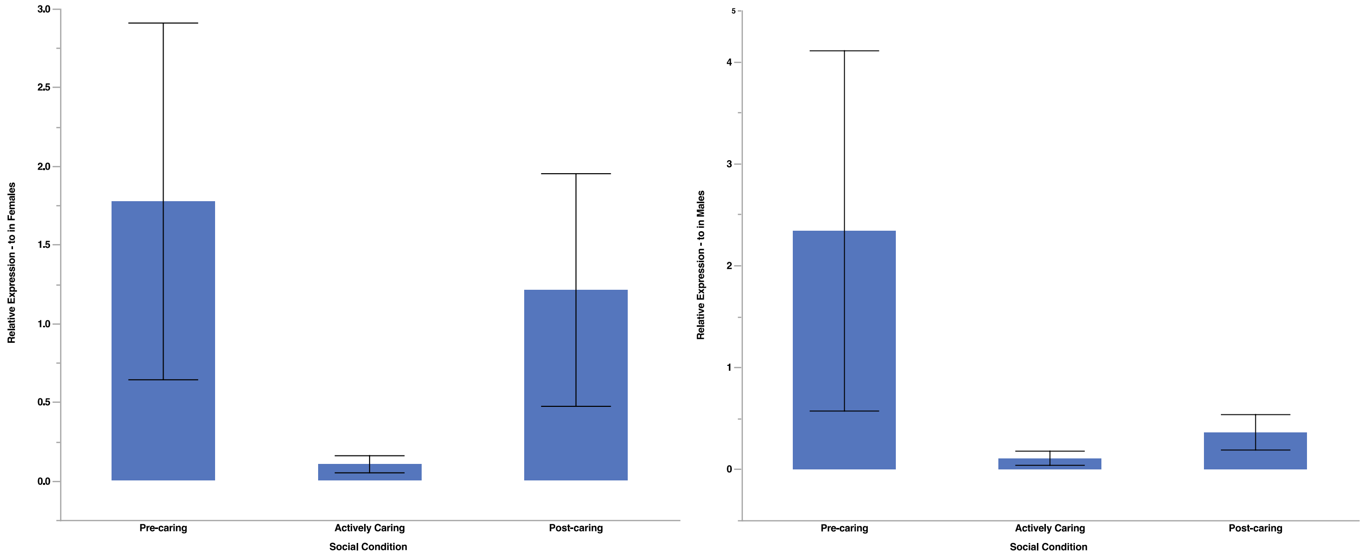

Supplementary Figure 12a | Relative expression of *takeout* (*to* under three behavioural stages – pre-caring (control, mated but not on a mouse); actively caring; post-caring (24 h after dispersal from the carcass by the caring female) in head tissue from **A)** uniparental female *Nicrophorus vespilloides*. ANVOA F=22.534, df = 2,27, P < 0.0001. Significance of pair-wise comparisons assessed by Fisher's LSD test: Pre-caring > caring, P < 0.0001; Pre-caring NS Post-caring, P = 0.826; Post-caring > caring, P = 0.0001. **B)** from uniparental male *N. vespilloides*. ANOVA F=13.325, df = 2,27, P < 0.0001. Significance of pair-wise comparisons assessed by Fisher's LSD test: Pre-caring > caring, P < 0.0001; Pre-caring > Post-caring, P = 0.014; Post-caring > caring, P = 0.018. Analyses performed on ln-transformed data ($-\Delta\Delta C_T$).). TATA-binding protein was used as an endogenous control, with 3 technical replicates and 10 biological replicates per gene and treatment. The pre-caring state was used as the baseline expression state.

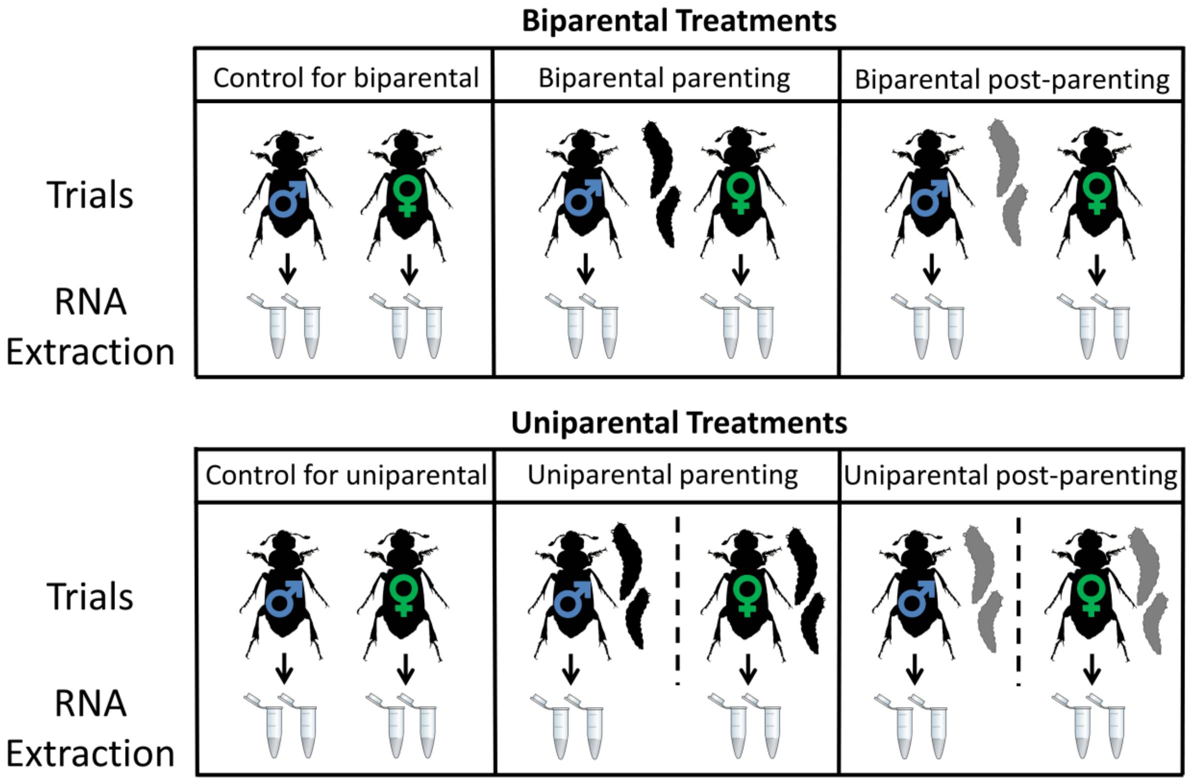

Supplementary Figure 13 | Outline of sample generation for transcriptomic analyses. Each beetle represents 20 individuals. These produced two biological replicate samples as the brains from 10 individuals were pooled for RNA extraction (represented by a microcentrifuge tube). Black larvae represent the presence of larvae, grey larvae represent larvae that have deserted. Beetle pairs were raised in 6 conditions: In both controls a male and female were allowed to mate but not provided with a mouse carcass and thus were unable to lay eggs and provide care. For the biparental parenting treatment a male and female pair were allowed to mate in the presence of a mouse carcass and allowed to reproduce and provide parental care. Biparental post-parenting samples were generated in the same way as parenting individuals, except they were collected after larvae had deserted. Uniparental parenting and post parenting samples were generated in the same way as biparental samples except one mate was removed post-pairing to leave the remaining mate in a uniparental condition

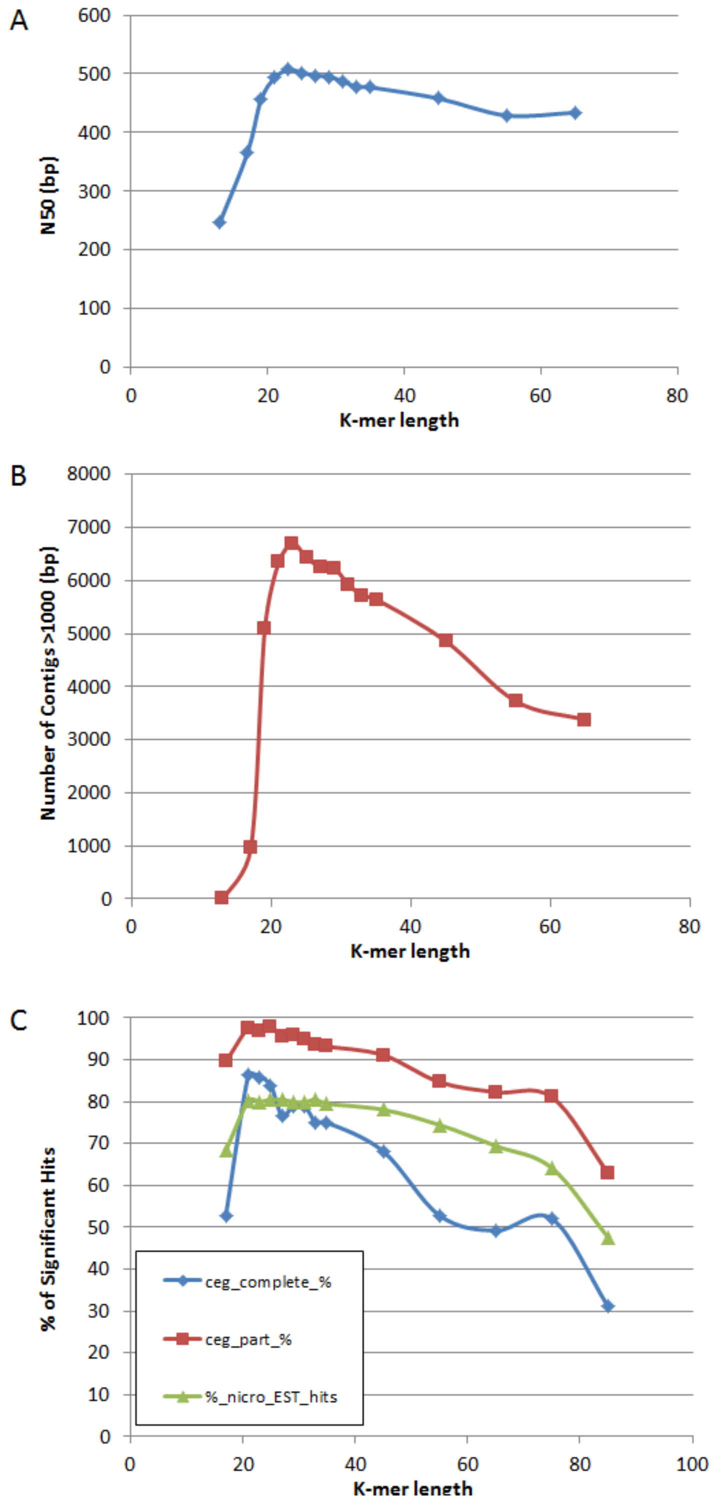

Supplementary Figure 14 | Assembly metrics for different k-mer lengths. A. N50 B. the number of contigs over 1000bp in the assembly C. Percentage of 'core eukaryotic genes' (both complete and partial) present in the assembly identified using the CEGMA pipeline[2] and the percentage of *Nicrophorus vespilloides* ESTs that had a significant blast hit in the assembly. ceg= core eukaryotic genes, nicro= *Nicrophorus vespilloides*, EST= expressed sequence tags, bp = base pairs

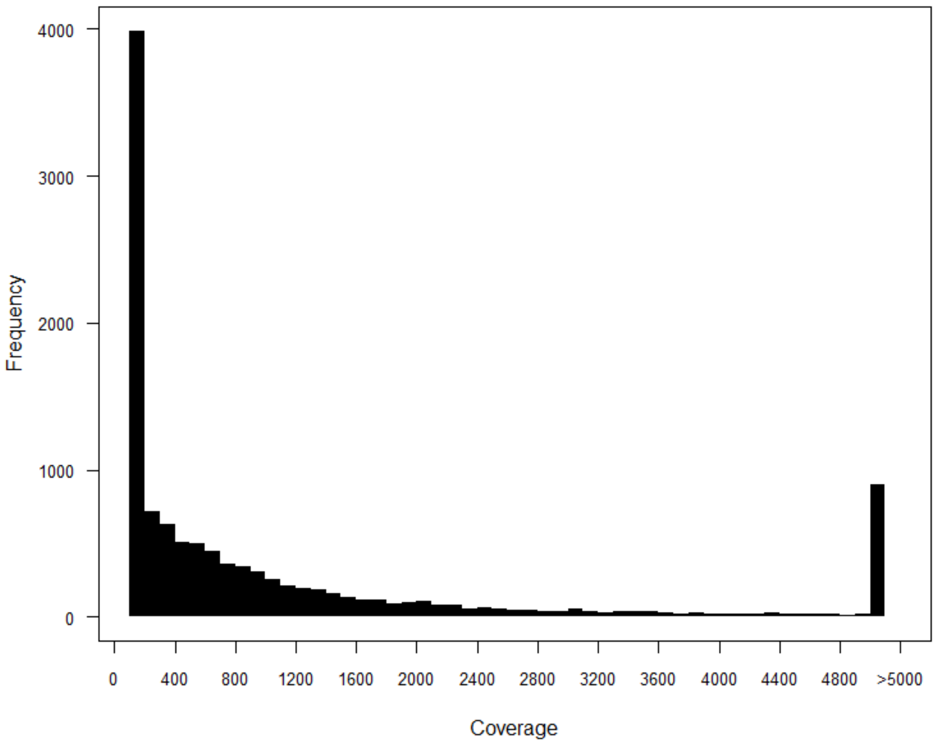

Supplementary Figure 15 | Mapping statistics. Mean coverage per scaffold for the reference assembly

Supplementary Table 1: The number of differentially expressed genes in the caring gene set in caring vs. control and post-caring vs. control comparisons using the "extended reference assembly".

Comparison	Parenting type	Males	Females
**Caring vs. control**	**Biparental**	30	776
	**Uniparental**	403	995
**Post-caring vs. control**	**Biparental**	28	42
	**Uniparental**	27	22

Supplementary Table 2 | The effect of sex, parental type, and caring state on gene expression change in the caring gene set when using the "extended reference assembly".

Variable	DF	Sum Sq	Mean Sq	F	*p*
**Sex**	1	433	433.4	305.457	$< 2 \times 10^{-16}$
**Uniparental/Biparental**	1	7	6.6	4.675	0.0306
**Caring/Non-caring**	1	1002	1002.4	706.461	$< 2 \times 10^{-16}$
**Sex * Parenting**	1	28	27.6	19.444	1.04×10^{-5}
**Sex * Caring**	1	223	222.7	156.985	$< 2 \times 10^{-16}$
**Parenting * Caring**	1	110	110.1	77.568	$< 2 \times 10^{-16}$
**Sex * Parenting * Caring**	1	8	7.5	5.301	0.0213
**Residual**	13176	18695	1.4		

Supplementary Table 3| Significantly enriched GO-terms in the caring gene set

GO-ID	Term	FDR
**GO:0006874**	cellular calcium ion homeostasis	6.59×10^{-10}
**GO:0005219**	ryanodine-sensitive calcium-release channel activity	1.28×10^{-09}
**GO:0005319**	lipid transporter activity	1.64×10^{-07}
**GO:0006869**	lipid transport	3.80×10^{-06}
**GO:0055114**	oxidation-reduction process	6.55×10^{-06}
**GO:0070588**	calcium ion transmembrane transport	8.37×10^{-06}
**GO:0016705**	oxidoreductase activity, acting on paired donors, with incorporation or reduction of molecular oxygen	4.98×10^{-05}
**GO:0020037**	heme binding	0.0001
**GO:0004252**	serine-type endopeptidase activity	0.0004
**GO:0005549**	odorant binding	0.0011
**GO:0042302**	structural constituent of cuticle	0.0014
**GO:0005506**	iron ion binding	0.0017
**GO:0005089**	Rho guanyl-nucleotide exchange factor activity	0.0026
**GO:0016849**	phosphorus-oxygen lyase activity	0.0028
**GO:0035023**	regulation of Rho protein signal transduction	0.0031
**GO:0009190**	cyclic nucleotide biosynthetic process	0.0050
**GO:0007601**	visual perception	0.0074
**GO:0019202**	amino acid kinase activity	0.0074
**GO:0016297**	acyl-[acyl-carrier-protein] hydrolase activity	0.0074
**GO:0080019**	fatty-acyl-CoA reductase (alcohol-forming) activity	0.0143
**GO:0018298**	protein-chromophore linkage	0.0185
**GO:0016459**	myosin complex	0.0286
**GO:0006026**	aminoglycan catabolic process	0.0323

Supplementary Table 4. Assembly and mapping statistics for *de novo* assembly (k=25) before (Raw assembly) and after filtering scaffolds for contamination (scaffolds that blasted to non-arthropod) or those that did not produce a significant blast hit (E-value > 0.001) to produce the reference assembly. Contig N50 and scaffold N50 were obtained using assemblethon[3].

Assembly	Total read pairs mapped (% of raw reads)	Size of transcriptome (bp)	mean coverage	Contig N50	scaffold N50	N scaffolds
Raw assembly	455949577 (63.9)	53919962	1522	1299	1884	48296
Reference assembly	430998221 (60.4)	35402209	2191	2110	2979	17019

Supplementary Table 5 Results from the CEGMA pipeline[2] for the reference assembly. Prots = number of 248 ultra-conserved CEGs (Core Eukaryotic Genes) present in genome, % Completeness = percentage of 248 ultra-conserved CEGs present, Total = total number of CEGs present including putative orthologs, Average = average number of orthologs per CEG, % Ortho = percentage of detected CEGS that have more than 1 ortholog.

	#Prots	%Completeness	#Total	Average	%Ortho
**Complete**	204	82.26	467	2.29	68.63
**Group 1**	49	74.24	123	2.51	75.51
**Group 2**	48	85.71	105	2.19	68.75
**Group 3**	54	88.52	126	2.33	72.22
**Group 4**	53	81.54	113	2.13	58.49
**Partial**	238	95.97	579	2.43	71.01
**Group 1**	63	95.45	166	2.63	79.37
**Group 2**	52	92.86	127	2.44	71.15
**Group 3**	59	96.72	140	2.37	69.49
**Group 4**	64	98.46	146	2.28	64.06

Supplementary Table 6 | Number of reads passing quality control (QC, quality trimming, removal of read pairs with adaptor sequences, removal of read pairs with reads <85bp) and number of reads mapping to reference assembly for each library.

Library number	Condition	sex	replicate	Raw Read pairs	Read pairs kept after QC	Read pairs kept after QC (%)	Read pairs mapped	Read pairs mapped (%)
1	control for biparental	Female	1	27198698	27186876	99.96	17729537	65.21
2	control for biparental	Female	2	26685779	26664998	99.92	18156673	68.09
3	control for uniparental	Female	1	30151331	29717574	98.56	19182562	64.55
4	control for uniparental	Female	2	30661662	30490233	99.44	20235243	66.37
5	control for biparental	Male	1	26307789	26291008	99.94	15660692	59.57
6	control for biparental	Male	2	25595478	25569149	99.90	14966333	58.53
7	control for uniparental	Male	1	29028894	28652974	98.71	17340698	60.52
8	control for uniparental	Male	2	30118973	29821098	99.01	18434455	61.82
9	Biparental parenting	Female	1	25580525	25556902	99.91	14647691	57.31
10	Biparental parenting	Female	2	27398893	27387771	99.96	15821030	57.77
11	Uniparental parenting	Female	1	27370110	27170276	99.27	16131079	59.37
12	Uniparental parenting	Female	2	35697769	35404437	99.18	21259071	60.05
13	Biparental parenting	Male	1	26725766	26702193	99.91	15457885	57.89
14	Biparental parenting	Male	2	26889467	26874825	99.95	15423028	57.39
15	Uniparental parenting	Male	1	39424372	39082745	99.13	23406818	59.89
16	Uniparental parenting	Male	2	40309706	39929606	99.06	23906173	59.87
17	Biparental post-parenting	Female	1	26170752	26142024	99.89	15583397	59.61
18	Biparental post-parenting	Female	2	25839070	25822740	99.94	15024559	58.18
19	Uniparental post-parenting	Female	1	36592277	36122310	98.72	23129177	64.03
20	Uniparental post-parenting	Female	2	31682815	31210473	98.51	19963728	63.96
21	Biparental post-parenting	Male	1	25588907	25563130	99.90	14776987	57.81
22	Biparental post-parenting	Male	2	26336968	26317536	99.93	15136007	57.51
23	Uniparental post-parenting	Male	1	35926096	34991857	97.40	20808150	59.47
24	Uniparental post-parenting	Male	2	30647493	30472720	99.43	18817248	61.75

Supplemental Table 7. Design matrix for the GLM used to identify DE genes. White columns - Sample information including factors Sex, Behavioural state, and Parental type provided to EdgeR. Grey columns = design matrix used in EdgeR to perform GLM and produce the contrasts listed in the main text. For more detailed information on the use of design matrix to perform contrasts see[4.]

Group	Replicate	Sex	behavioural state	Parental type	Sex	Parenting	Post-parenting	Parental type	Sex: Parenting	Sex: Post-parenting	Sex: Parental Type	Parenting: Parental type	Post-parenting: Parental Type	Sex: Parenting: Parental	Sex: Post-parenting: Parental type
biparental female parenting	1	female	parenting	biparental	0	1	0	0	0	0	0	0	0	0	0
biparental female parenting	2	female	parenting	biparental	0	1	0	0	0	0	0	0	0	0	0
uniparental female parenting	1	female	parenting	uniparental	0	1	0	1	0	0	0	1	0	0	0
uniparental female parenting	2	female	parenting	uniparental	0	1	0	1	0	0	0	1	0	0	0
biparental female control	1	female	control	biparental	0	0	0	0	0	0	0	0	0	0	0
biparental female control	2	female	control	biparental	0	0	0	0	0	0	0	0	0	0	0
uniparental female control	1	female	control	uniparental	0	0	0	1	0	0	0	0	0	0	0
uniparental female control	2	female	control	uniparental	0	0	0	1	0	0	0	0	0	0	0
biparental female post-parenting	1	female	post-parenting	biparental	0	0	1	0	0	0	0	0	0	0	0
biparental female post-parenting	2	female	post-parenting	biparental	0	0	1	0	0	0	0	0	0	0	0
uniparental female post-parenting	1	female	post-parenting	uniparental	0	0	1	1	0	0	0	0	1	0	0
uniparental female post-parenting	2	female	post-parenting	uniparental	0	0	1	1	0	0	0	0	1	0	0
biparental male parenting	1	male	parenting	biparental	1	1	0	0	1	0	0	0	0	0	0
biparental male parenting	2	male	parenting	biparental	1	1	0	0	1	0	0	0	0	0	0
uniparental male parenting	1	male	parenting	uniparental	1	1	0	1	1	0	1	1	0	1	0
uniparental male parenting	2	male	parenting	uniparental	1	1	0	1	1	0	1	1	0	1	0
biparental male control	1	male	control	biparental	1	0	0	0	0	0	0	0	0	0	0
biparental male control	2	male	control	biparental	1	0	0	0	0	0	0	0	0	0	0
uniparental male control	1	male	control	uniparental	1	0	0	1	0	0	1	0	0	0	0

uniparental male control	2	male	control	uniparental	1	0	0	1	0	0	1	0	0	0	0
biparental male post-parenting	1	male	post-parenting	biparental	1	0	1	0	0	1	0	0	0	0	0
biparental male post-parenting	2	male	post-parenting	biparental	1	0	1	0	0	1	0	0	0	0	0
uniparental male post-parenting	1	male	post-parenting	uniparental	1	0	1	1	0	1	1	0	1	0	1
uniparental male post-parenting	2	male	post-parenting	uniparental	1	0	1	1	0	1	1	0	1	0	1

Supplementary References

1. Roy-Zokan, E. M., Cunningham, C. B., Hebb, L. E., McKinney, E. C. & Moore, A. J. *vitellogenin* and vitellogenin receptor gene expression is associated with male and female parenting in a subsocial insect. *Proc. R. Soc. Lond. B Biol. Sci.* **282,** 20150787 (2015).
2. Parra, G., Bradnam, K. & Korf, I. CEGMA: a pipeline to accurately annotate core genes in eukaryotic genornes. *Bioinformatics* **23,** 1061–1067 (2007).
3. Bradnam, K. R. *et al.* Assemblathon 2: evaluating de novo methods of genome assembly in three vertebrate species. *GigaScience* **2,** 10 (2013).
4. Chen, Y., McCarthy, D., Robinson, M. & Smyth, G. K. *edgeR: differential expression analysis of digital gene expression data User's Guide*. (2015). <http://www.bioconductor.org/packages/release/bioc/vignettes/edgeR/inst/doc/edgeRUsersGuide.pdf>
